# Salicylic Acid as a Food‐Derived Bioactive Constituent of Tiaoqi Daozhi Decoction: A Potential Functional Food Ingredient to Restoring Gut Barrier Function in Constipation‐Predominant IBS

**DOI:** 10.1002/fsn3.72483

**Published:** 2026-10-26

**Authors:** Min Duan, Lixia Huang, Yingyue Xu, Ya Huang, Xiaojun Yang

**Affiliations:** ^1^ Department of Gastroenterology Chongqing Hospital of Traditional Chinese Medicine Chongqing China; ^2^ Chongqing Hospital of Traditional Chinese Medicine Chongqing China

**Keywords:** functional food, gut barrier protection, IBS‐C, medicine‐food homology, metabolomics, salicylic acid, Tiaoqi Daozhi Decoction

## Abstract

Constipation‐predominant irritable bowel syndrome (IBS‐C) is a chronic gut disorder with limited dietary management options. Tiaoqi Daozhi Decoction (TQDZD), composed of medicine‐food homologous plant materials traditionally consumed for digestive comfort, represents a dietary strategy with functional food potential, yet its systemically bioavailable constituents and nutritional mechanisms remain unclear. This study aimed to characterize the orally bioavailable polyphenol profile of TQDZD and elucidate its barrier‐protective mechanisms within a functional food framework. Using ultra‐performance liquid chromatography–tandem mass spectrometry (UPLC‐MS/MS) serum metabolite profiling, we characterized TQDZD's polyphenol composition and identified orally bioavailable constituents in rat circulation after dietary administration. Untargeted serum metabolomics coupled with orthogonal partial least‐squares discriminant analysis (OPLS‐DA) was performed to map metabolic network perturbations in an IBS‐C rat model, and dietary bioactive pathways were predicted by comparing intervention‐induced metabolic trajectories. IBS‐C was induced in rats by ice‐water enema, and an in vitro inflammatory model was established using lipopolysaccharide (LPS)‐stimulated Caco‐2 monolayers. Barrier function was assessed by fluorescein isothiocyanate (FITC)‐dextran flux; tight junction proteins, cytokines, and signaling pathways were evaluated by Western blot, immunofluorescence, ELISA, and qPCR. To explore the mechanistic basis of the observed barrier‐protective effects, Bruton's tyrosine kinase (BTK) signaling involvement was assessed using siRNA‐mediated knockdown and plasmid‐mediated overexpression as complementary pharmacological tools. A total of 1962 non‐redundant compounds were identified in TQDZD aqueous extract, with phenolic acids and flavonoids as the predominant chemical classes. These polyphenol‐rich profiles are characteristic of functional foods with gut‐protective potential. In dosed rat serum, 257 systemically bioavailable components were detected, of which 150 (58.4%) were prototype constituents. Multi‐criteria screening (treatment/control peak area ratio ≥ 2, top‐20 abundance rank in the formula, coefficient of variation < 30%, and Level 1 identification confidence) yielded four core prototypes: salicylic acid, N‐acetyl‐L‐phenylalanine, L‐valyl‐L‐phenylalanine, and L‐phenylalanyl‐L‐hydroxyproline. Among these, salicylic acid—a naturally occurring phenolic acid widely distributed in plant‐based foods—was prioritized for functional validation. Untargeted metabolomics revealed that IBS‐C rats exhibited profound dysregulation of a lipid‐inflammatory network, characterized by disturbances in glycerophospholipid metabolism, linoleic acid metabolism, and sphingolipid signaling. TQDZD intervention remodeled this network in a dose‐dependent manner; notably, the metabolic trajectory of the high‐dose TQDZD group converged strikingly with that of the BTK modulator orelabrutinib. In vivo, TQDZD significantly reduced intestinal permeability (*p* < 0.001), restored colonic expression of tight junction proteins zonula occludens‐1 (ZO‐1), Occludin, and Claudin‐1 (*p* < 0.001), and suppressed mucosal and serum levels of tumor necrosis factor‐α (TNF‐α), interleukin‐6 (IL‐6), IL‐1β, and monocyte chemoattractant protein‐1 (MCP‐1) (*p* < 0.001). Mechanistic studies demonstrated that Tiaoqi Daozhi Decoction (TQDZD) inhibited the BTK‐spleen tyrosine kinase (SYK)‐ phospholipase C gamma 2 (PLCγ2)‐protein kinase B (AKT) axis, thereby blocking IκBα phosphorylation and p65 nuclear translocation. BTK knockdown potentiated, whereas BTK overexpression abrogated, the barrier‐protective and anti‐inflammatory effects of TQDZD. Salicylic acid alone fully reproduced TQDZD's phenotype in vitro and in vivo, and its effects were strictly BTK‐dependent. TQDZD exerts its therapeutic effect on constipation‐predominant irritable bowel syndrome (IBS‐C) via its core systemically bioavailable constituent salicylic acid. The biological activity of salicylic acid relies on the BTK signaling pathway, which blocks the non‐canonical nuclear factor‐κB (NF‐κB) pathway, thereby restoring the intestinal epithelial barrier and suppressing low‐grade inflammation. Given salicylic acid's abundance in edible plants and its long history of safe dietary exposure, these findings provide a mechanistic rationale for developing phenolic‐acid‐rich botanical blends as supportive dietary strategies for IBS‐C and related barrier‐centric gastrointestinal disorders.

## Graphical abstract

Tiaoqi Daozhi Decoction (TQDZD) is a classic formulation composed of eight medicine‐food homologous botanicals. UPLC‐MS/MS profiling identified 1962 non‐redundant compounds in its aqueous extract, predominantly phenolic acids and flavonoids, of which 107 metabolites were systemically bioavailable. Through multi‐dimensional screening and in vivo/in vitro validation, salicylic acid (SA) was identified as the core systemically bioavailable active constituent. SA inhibits the BTK signaling pathway, blocking SYK/PLCγ2/AKT phosphorylation and the downstream IκBα/p65 non‐canonical NF‐κB cascade, thereby preventing p65 nuclear translocation. This suppresses the release of TNF‐α, IL‐6, IL‐1β, and MCP‐1, and attenuates mast cell degranulation and CD19^+^ B cell infiltration. Concurrently, SA upregulates the tight junction proteins ZO‐1, Occludin, and Claudin, reduces FITC‐dextran permeability, and restores intestinal epithelial barrier integrity, ultimately ameliorating the low‐grade inflammation and barrier dysfunction characteristic of constipation‐predominant irritable bowel syndrome (IBS‐C).

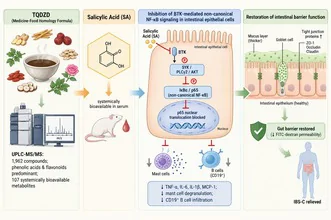

## 1 Introduction

Irritable bowel syndrome (IBS) is a prevalent functional gastrointestinal disorder worldwide, with the constipation‐predominant subtype (IBS‐C) accounting for approximately one‐third of cases (Oka et al. 2020). Characterized by chronic abdominal pain and altered bowel habits dominated by constipation, IBS‐C substantially diminishes patients' quality of life and imposes a considerable healthcare burden. Current pharmacotherapies, including antispasmodics, osmotic laxatives, and secretagogues, offer only symptomatic palliation with limited long‐term efficacy, and relapse following treatment discontinuation is common (Ye et al. 2026). In recent years, there has been growing interest in dietary strategies and functional foods as safe, sustainable adjunctive approaches for managing chronic gastrointestinal conditions (Hossain et al. 2025; Marcìa‐Fuentes et al. 2026; Ma et al. 2026). This interest is deeply rooted in the ancient philosophy of “medicine and food homology” (yao shi tong yuan), which holds that many edible plants possess both nutritional value and bioactive properties capable of modulating physiological functions (Wang et al. 2025; Liu et al. 2025; He et al. 2024). This concept is increasingly recognized by modern nutritional science as a framework for developing evidence‐based functional food ingredients, particularly in the context of gut health (Lv et al. 2024; Law and Au 2026; Yang et al. 2023), where food‐derived bioactive compounds are being explored for their potential to reinforce epithelial barrier integrity, modulate gut microbiota, and mitigate low‐grade inflammation through multi‐target, moderate‐affinity interactions compatible with chronic dietary intake.

The pathogenesis of IBS‐C is multifactorial, involving perturbations of the brain–gut axis, increased intestinal mucosal permeability, low‐grade mucosal inflammation, and immune dysregulation (Sperber et al. 2021; Videlock and Chang 2021). Among these, chronic low‐grade inflammation has emerged as a convergence point that mediates crosstalk between the enteric nervous system and the intestinal epithelial barrier, linking sensory abnormalities to barrier dysfunction (Rawla et al. 2018). Notably, dietary polyphenols, particularly phenolic acids and flavonoids abundant in plant‐based foods, have shown promise in preclinical models for modulating these pathological processes, offering a nutritional approach to restoring intestinal homeostasis (Saad et al. 2025; Kumar and Goel 2019). However, current clinical agents do not adequately address these underlying defects (Ferreira et al. 2020). This gap has prompted the exploration of functional food ingredients, particularly those derived from botanical sources with a history of culinary and medicinal use, as promising candidates for restoring intestinal homeostasis. Such ingredients offer the distinct advantage of high safety profiles, making them suitable for long‐term dietary incorporation.

Tiaoqi Daozhi Decoction (TQDZD) is a classic multi‐herbal formulation traditionally used for regulating gastrointestinal motility and alleviating stagnation. Its constituent botanicals, including Cyperi Rhizoma (nutgrass galingale rhizome), Linderae Radix (spicebush root), *Cannabis Sativa* L. (hemp seed), and *Amygdalus Communis* Vas (sweet almond), are noteworthy not only for their therapeutic legacy but also for their status as edible or food‐grade materials (Han et al. 2021). Several of these are listed in both the Chinese Pharmacopeia and the official Catalogue of Medicinal and Food Homologous Substances, reflecting their long‐standing dual use as food and medicine and positioning TQDZD as a candidate functional food rather than a pharmaceutical agent (Li et al. 2017). In clinical practice, TQDZD has demonstrated notable efficacy in alleviating IBS‐C symptoms, yet its active constituents and molecular mechanisms have remained largely undefined, a common challenge in the modernization of traditional botanical mixtures into standardized functional food products. Given that preliminary phytochemical profiling has revealed phenolic acids and flavonoids as its predominant chemical classes, we reasoned that TQDZD may serve as a rich source of dietary polyphenols with barrier‐protective and anti‐inflammatory activities.

From a pathophysiological perspective, the Bruton's tyrosine kinase (BTK)–spleen tyrosine kinase (SYK)–nuclear factor kappa B (NF‐κB) signaling axis acts as a core molecular driver of IBS‐C progression. Aberrant activation of this pathway directly disrupts tight junction architecture, elevates intestinal mucosal permeability, and triggers sustained release of pro‐inflammatory cytokines, which together maintain the chronic low‐grade inflammatory state and epithelial barrier damage that are hallmarks of IBS‐C (Neys et al. 2021; Yang et al. 2024; Robak and Robak 2022). Targeting this pathological cascade has therefore been proposed as a promising strategy to reverse the underlying lesions of IBS‐C, rather than merely relieving symptoms.

BTK is a non‐receptor tyrosine kinase that plays a pivotal role in innate and adaptive immune signaling, particularly through its regulation of the SYK‐NF‐κB axis and subsequent release of pro‐inflammatory cytokines (Guan et al. 2021; Tavakoli et al. 2024; Rozkiewicz et al. 2023). While BTK is well‐characterized as a pharmacological target, emerging evidence suggests that dietary polyphenols can modulate tyrosine kinase signaling through non‐competitive, allosteric, or metabolite‐mediated mechanisms, offering a milder regulatory profile compatible with long‐term dietary intake. Naturally occurring BTK‐modulating compounds derived from edible botanicals therefore represent an attractive alternative to selective inhibitors, providing balanced immunoregulation without the systemic immunosuppression associated with pharmaceutical agents. However, whether food‐borne bioactive constituents from TQDZD can engage this pathway to alleviate IBS‐C remains unexplored.

Against this backdrop, the present study was designed to establish a mechanistic link between the systemically bioavailable constituents of TQDZD, the immunometabolic network, and intestinal barrier function, framed within the context of functional food science and the medicine‐food homology paradigm. Using high‐resolution UPLC‐MS/MS, we identified orally bioavailable components and, through untargeted serum metabolomics, delineated the metabolic perturbations in a rat model of IBS‐C. We further evaluated the effects of TQDZD on intestinal permeability, tight junction protein expression, and inflammatory cytokine levels in vivo, and employed BTK knockdown and overexpression strategies in vitro to dissect the role of BTK signaling in mediating the barrier‐protective and anti‐inflammatory actions of the formula. Finally, the core constituent identified from screening was validated in both animal and cellular models to confirm its BTK‐dependent effects. We hypothesized that TQDZD, via its core dietary bioactive constituent, modulates the BTK‐SYK‐NF‐κB signaling cascade, thereby remodeling the lipid‐driven inflammatory metabolic network, alleviating low‐grade mucosal inflammation, restoring epithelial barrier integrity, and ultimately conferring protective benefits in IBS‐C through a functional food‐compatible mechanism. This work not only elucidates the mechanism of a traditional botanical formula but also provides a scientific rationale for its development as a functional food ingredient targeting intestinal health.

## 2 Materials and Methods

### 2.1 Animal Procedures and Sample Collection

Male Sprague–Dawley rats (specific pathogen‐free, 180–200 g) were obtained from Chongqing Ensiweier Biotechnology Co. Ltd. Animals were housed under controlled conditions (22°C ± 2°C, 50% ± 5% humidity, 12 h light/dark cycle) with free access to food and water, and were allowed to acclimatize for 7 days prior to experimentation. All animal procedures were approved by the Institutional Animal Care and Use Committee (IACUC) of Southwest University (approval number: IACUC‐20260312‐13).

Tiaoqi Daozhi Decoction (TQDZD) was composed of the following medicine‐food homologous plant materials (10 g each): Cyperi Rhizoma, Linderae Radix, *Cannabis Sativa* L., Amygdalus Communis Vas, Rose Rugosae Flos, Trichosanthes Kirilowii Maxim, Curcumae Rhizoma, and Menthae Herba. For systemic bioavailability analysis of dietary polyphenols, six rats were randomly allocated to a blank group (*n* = 3) and a TQDZD‐treated group (*n* = 3). Treated rats received a single oral administration of TQDZD aqueous extract (crude drug concentration 1 g/mL, 10 mL/kg body weight), whereas blank controls received an equal volume of normal saline. One hour post‐administration (the time to peak plasma concentration determined in pilot experiments), rats were anesthetized by intraperitoneal injection of 3% sodium pentobarbital (30 mg/kg). Blood (5 mL) was collected from the abdominal aorta into anticoagulant‐free tubes, allowed to clot at room temperature for 30 min, and centrifuged at 3000 rpm for 15 min at 4°C. Serum was aliquoted and stored at −80°C. The original TQDZD aqueous extract was retained as a reference sample.

For in vivo intervention studies, 70 rats were randomly assigned to seven groups (*n* = 10 per group): normal control (NC), IBS‐C model (MC), pinaverium bromide reference (P, 15 mg/kg), orelabrutinib reference (Y, 10 mg/kg), and TQDZD low‐ (ML, 2.5 g crude material/kg), medium‐ (MM, 5 g crude material/kg), and high‐dose (MH, 10 g crude material/kg) groups. The IBS‐C model was induced by ice‐water gavage combined with restraint stress: all groups except NC received daily intragastric administration of ice‐cold water (0°C–4°C, 10 mL/kg) followed by 1 h restraint in a custom‐built apparatus for 14 consecutive days. This protocol reproducibly induces colonic low‐grade inflammation and barrier dysfunction, mimicking key features of human IBS‐C. NC rats received an equivalent volume of room‐temperature normal saline without restraint. Following model induction, TQDZD or reference compounds were orally delivered once daily for 14 d as part of a dietary intervention regimen; NC and MC groups received normal saline. Twenty‐four hours after the final intervention, rats were fasted for 12 h with free access to water, then anesthetized with 3% sodium pentobarbital. Blood (5 mL) was collected from the abdominal aorta: 2 mL was placed in EDTA (ethylenediaminetetraacetic acid)‐coated tubes and centrifuged (3000 rpm, 10 min, 4°C) to obtain plasma for intestinal permeability assays; 3 mL was collected into anticoagulant‐free tubes and processed identically to obtain serum, which was aliquoted and stored at −80°C for untargeted metabolomics and ELISA. Rats were euthanized by intraperitoneal injection of 120 mg/kg sodium pentobarbital. The distal colon (2 cm) was rapidly excised and divided into three portions: one fixed in 4% paraformaldehyde for histological and immunohistochemical analyses; one snap‐frozen in liquid nitrogen and stored at −80°C for Western blotting and qPCR; and the remainder stored at −80°C as backup. The IBS‐C rat model established by ice water gavage combined with restraint stress has been fully validated in previous studies. It can stably induce typical IBS‐C phenotypes, including attenuated intestinal motility, reduced fecal output, and low‐grade colonic mucosal inflammation (Zhu et al. 2010; Rushikesh et al. 2026; Zou et al. 2008).

### 2.2 Chemical Characterization and Systemic Bioavailability Assessment of TQDZD Polyphenols

Qualitative analysis was performed on a UPLC‐Q Exactive HF‐X high‐resolution mass spectrometry platform. Chromatography‐grade methanol and acetonitrile were purchased from Merck, and formic acid from Aladdin. Separation was achieved on a Waters ACQUITY Premier HSS T3 column (1.8 μm, 2.1 × 100 mm) with mobile phase A (0.1% formic acid in water) and B (0.1% formic acid in acetonitrile). The gradient was: 0–2 min, 5%–25% B; 2–3 min, 25%–35% B; 3–5 min, 35%–60% B; 5–6 min, 60%–99% B, held for 1.5 min; 7.6 min, return to 5% B and equilibration to 10 min. Flow rate: 0.35 mL/min; column temperature: 40°C; injection volume: 4 μL. Mass spectrometry employed electrospray ionization in both positive and negative ion modes (spray voltage: 3500 V and 3200 V, respectively; ion transfer tube temperature: 320°C; vaporizer temperature: 300°C). MS^1^ spectra were acquired at 35,000 resolution over m/z 84–1250; MS^2^ spectra were acquired at 17,500 resolution with stepped collision energies (30, 40, 50 eV) and a dynamic exclusion time of 3 s.

TQDZD aqueous extract was lyophilized (Scientz‐100F), pulverized using an MM 400 grinder (30 Hz, 1.5 min), and 30 mg of powder was extracted with 1.5 mL ice‐cold 70% methanol containing 1 ppm internal standard. The mixture was vortexed for 30 s every 30 min over six cycles, centrifuged at 12,000 rpm for 3 min, and the supernatant was filtered through a 0.22 μm organic membrane prior to injection. Rat serum samples were thawed on ice; 100 μL serum was mixed with 500 μL 20% acetonitrile in methanol containing internal standards, vortexed for 3 min, and centrifuged at 12,000 rpm for 10 min at 4°C. After standing at −20°C for 30 min, 470 μL of supernatant was dried under nitrogen and reconstituted in 200 μL 70% methanol. Following a final centrifugation, the supernatant was injected. Raw data were converted to mzML format using ProteoWizard. Peak detection, alignment, and retention time correction were performed with XCMS. Peaks with > 50% missing values were filtered out, and peak areas were normalized using support vector regression. Metabolite identification was based on matching against an in‐house authentic standard library, the HMDB (Human Metabolome Database) public database, and a TCM (traditional Chinese medicine) metabolite database, retaining only compounds with a composite score > 0.5. Systemically bioavailable prototype constituents were defined as those present in both the original decoction and serum following dietary intake, with a peak area ratio (treatment/control) ≥ 2. Systemically bioavailable metabolites were defined as those detected exclusively in dosed serum, exhibiting Phase I/II metabolic characteristics, and meeting the same ratio threshold. Extracted ion chromatograms and MS/MS spectra of core constituents were subjected to triple confirmation based on retention time, accurate mass, and characteristic fragment ions.

### 2.3 Untargeted Serum Metabolomics Analysis

This analysis included the normal control (NC), IBS‐C model (MC), pinaverium bromide reference (P), orelabrutinib reference (Y), and TQDZD low‐ (ML), medium‐ (MM), and high‐dose (MH) groups, with three rats per group. Serum samples stored at −80°C were thawed on ice, and 50 μL aliquots were mixed with 300 μL of 20% acetonitrile–methanol containing internal standards (chromatography‐grade methanol and acetonitrile, Merck). After vortexing for 3 min, samples were centrifuged at 12,000 rpm for 10 min at 4°C. A 200 μL portion of the supernatant was held at −20°C for 30 min and centrifuged again under the same conditions for 3 min; 180 μL of the final supernatant was transferred to vial inserts for analysis. A pooled quality control (QC) sample was inserted after every ten experimental samples to monitor analytical reproducibility throughout the run.

Chromatographic separation was performed on a Thermo Vanquish ultra‐high‐performance liquid chromatography (UHPLC) system coupled to a Q Exactive HF‐X high‐resolution mass spectrometer. A Waters ACQUITY Premier HSS T3 column (1.8 μm, 2.1 × 100 mm) was used with mobile phase A (0.1% formic acid in water; formic acid from Aladdin) and B (0.1% formic acid in acetonitrile). The gradient was: 0–1 min, 5%–20% B; 1–3 min, 20%–99% B, held for 1.5 min; 4.6 min, return to 5% B and equilibration to 6 min. Flow rate: 0.4 mL/min; column temperature: 40°C; injection volume: 3 μL. Mass spectrometry employed electrospray ionization in both positive and negative ion modes (spray voltage: 3800 V and 3400 V, respectively; ion transfer tube temperature: 320°C; vaporizer temperature: 350°C positive, 650°C negative). Sheath gas and auxiliary gas were set to 60 and 20 arbitrary units, respectively. MS^1^ spectra were acquired at 60,000 resolution over m/z 70–1000 with an AGC target of 3.0 × 10^6^; MS^2^ spectra were acquired at 15,000 resolution with stepped collision energies (30, 40, 50 eV), an AGC target of 1.0 × 10^5^, and a dynamic exclusion time of 3 s.

Raw data were converted to mzML format using ProteoWizard. Peak detection, alignment, and retention time correction were performed with the XCMS package. Peaks with > 50% missing values within any group were filtered; remaining missing values were imputed by K‐nearest neighbor (< 50% missing) or one‐fifth minimum value (≥ 50% missing) approaches. Peak areas were normalized using support vector regression. Only metabolites with QC sample coefficient of variation (CV) < 0.3 and composite identification score > 0.5 were retained. Metabolite annotation was performed by matching against an in‐house library, public databases, and predictive spectral libraries; redundant features across ionization modes were merged. All statistical analyses were conducted in R (v4.3.1). Peak area data were log_2_‐transformed and subjected to unit variance (UV) scaling. Unsupervised principal component analysis (PCA) was initially performed to assess within‐group clustering and between‐group separation. Supervised orthogonal partial least‐squares discriminant analysis (OPLS‐DA) was subsequently applied using MetaboAnalystR (v1.0.1) to maximize inter‐group discrimination. Model reliability was validated by seven‐fold cross‐validation and 200 permutation tests to exclude overfitting (criteria: R^2^Y > 0.8 and Q^2^ > 0.5).

Differential metabolites were identified by integrating multivariate and univariate statistical criteria: variable importance in projection (VIP) > 1 from OPLS‐DA, together with Benjamini–Hochberg adjusted *p* < 0.05 from two‐tailed Student's *t*‐test (two‐group comparisons) or one‐way ANOVA (multi‐group comparisons). Volcano plots were generated using ggplot2 (v3.4.4) to visualize fold‐change and statistical significance between groups. Heatmaps of differential metabolites were constructed with ComplexHeatmap (v2.14.0) following row‐wise UV scaling, with bidirectional hierarchical clustering based on Euclidean distance and the ward.D2 linkage method. Pearson correlation networks of differential metabolites were built using igraph (v1.2.11), applying thresholds of |*r*| > 0.7 and false discovery rate (FDR) < 0.05. Kyoto Encyclopedia of Genes and Genomes (KEGG) (v108.0) pathway enrichment analysis was performed with the clusterProfiler package (v4.8.3). Enrichment significance was calculated by hypergeometric test, and the Rich Factor (number of differential metabolites/total annotated metabolites in the pathway) was used to indicate enrichment extent. Pathways with Benjamini–Hochberg adjusted *p* < 0.05 were considered significantly enriched. Bubble plots were generated using ggplot2 to display significantly enriched pathways.

### 2.4 Cell Culture and Experimental Groups

The human colorectal adenocarcinoma Caco‐2 cell line was obtained from the Cell Bank of the Chinese Academy of Sciences (Shanghai, China). Cells were cultured in high‐glucose Dulbecco's Modified Eagle Medium (DMEM) (Gibco, Cat. No. 11965092) supplemented with 10% fetal bovine serum (Gibco, Cat. No. 10099141) and 1% penicillin–streptomycin (Gibco, Cat. No. 15140122) at 37°C in a humidified incubator with 5% CO_2_. Cells were passaged every 2–3 days.

Caco‐2 cells were seeded into appropriate culture plates and allowed to reach 70%–80% confluence prior to transfection. Transfections were performed using Lipofectamine 3000 reagent (Invitrogen, Cat. No. L3000015) with BTK‐specific siRNA, negative control siRNA (si‐NC), a BTK overexpression plasmid (OE‐BTK), or an empty vector control plasmid (OE‐NC). Twenty‐four hours post‐transfection, cells were stimulated with 1 μg/mL lipopolysaccharide (LPS; Sigma‐Aldrich, Cat. No. L2880) for 24 h to establish an intestinal epithelial inflammation model. Concurrently, cells were treated with TQDZD‐containing serum (10%, v/v), salicylic acid (100 μmol/L), or ibrutinib (1 μmol/L). Eight experimental groups were defined: normal control (NC), LPS stimulation alone, LPS + TQDZD/salicylic acid, LPS + BTK reference modulator (ibrutinib), si‐BTK + LPS + TQDZD/salicylic acid, si‐NC + LPS + TQDZD/salicylic acid, OE‐BTK + LPS + TQDZD/salicylic acid, and OE‐NC + LPS + TQDZD/salicylic acid.

### 2.5 Permeability Assays

#### 2.5.1 Intestinal Epithelial Permeability In Vivo

Twenty‐four hours after the final intervention, rats were fasted for 12 h with free access to water and then received an oral gavage of 4 kDa FITC–dextran (Sigma‐Aldrich, Cat. No. FD4) at 400 mg/kg body weight. Four hours later, rats were anesthetized by intraperitoneal injection of 3% sodium pentobarbital (30 mg/kg), and blood was collected from the abdominal aorta. Serum was separated by centrifugation at 3000 rpm for 10 min at 4°C. Fluorescence intensity was measured using a multimode microplate reader (Tecan Infinite M200 Pro) at excitation and emission wavelengths of 490 nm and 520 nm, respectively. FITC–dextran concentrations were calculated from a standard curve.

#### 2.5.2 Monolayer Permeability In Vitro

Caco‐2 cells were seeded onto Transwell inserts (Corning, Cat. No. 3401) and cultured for 21 days until a confluent monolayer with transepithelial electrical resistance (TEER) > 1000 Ω·cm^2^ was achieved. Following treatments as described above, 100 μg/mL FITC–dextran was added to the apical compartment, and serum‐free medium was added to the basolateral compartment. After 2 h incubation, basolateral medium was collected, and fluorescence intensity was measured as above to assess monolayer permeability.

### 2.6 Histological Staining

Distal colon segments (2 cm) were fixed in 4% paraformaldehyde for 24 h, routinely dehydrated, cleared, and embedded in paraffin. Sections (4 μm) were prepared and subjected to hematoxylin and eosin (HE) staining (Beyotime, Cat. No. C0105), periodic acid–Schiff (PAS) staining (Sigma‐Aldrich, Cat. No. 395B), and toluidine blue staining (Solarbio, Cat. No. G1360). Colonic mucosal morphology, goblet cell abundance, and mast cell infiltration/degranulation were examined under a light microscope (Olympus BX53).

### 2.7 Immunohistochemistry

Paraffin sections were deparaffinized and rehydrated. Endogenous peroxidase activity was blocked with 3% H_2_O_2_ for 10 min at room temperature. Antigen retrieval was performed by boiling in citrate buffer (pH 6.0) under high pressure for 15 min. Sections were blocked with 5% bovine serum albumin (BSA) for 1 h at room temperature and then incubated overnight at 4°C with rabbit anti‐rat tryptase alpha/beta 1 (TPSAB1) primary antibody (Abcam, Cat. No. ab231516, 1:200) or rabbit anti‐rat CD19 primary antibody (Cell Signaling Technology, Cat. No. 90176, 1:200). The following day, sections were incubated with HRP‐conjugated goat anti‐rabbit secondary antibody (ZSGB‐BIO, Cat. No. ZB‐2301, 1:500) for 1 h at room temperature. Immunoreactivity was visualized using DAB (3,3′‐diaminobenzidine) substrate, and sections were counterstained with hematoxylin, dehydrated, cleared, and mounted. Images were acquired using a light microscope.

### 2.8 Immunofluorescence Staining

Frozen colonic tissue sections (5 μm) were fixed in 4% paraformaldehyde for 15 min, permeabilized with 0.3% Triton X‐100 for 10 min, and blocked with 5% BSA for 1 h at room temperature. Sections were incubated overnight at 4°C with rabbit anti‐rat ZO‐1 (Invitrogen, Cat. No. 40–2200, 1:200), rabbit anti‐rat Occludin (Invitrogen, Cat. No. 71–1500, 1:200), or rabbit anti‐rat Claudin‐1 (Invitrogen, Cat. No. 51–9000, 1:200) primary antibodies. The next day, sections were incubated with Alexa Fluor 488‐conjugated goat anti‐rabbit secondary antibody (Invitrogen, Cat. No. A11008, 1:500) for 1 h at room temperature in the dark. Nuclei were counterstained with DAPI (4′,6‐diamidino‐2‐phenylindole), and sections were mounted with anti‐fade mounting medium. Images were captured using a laser scanning confocal microscope (Zeiss LSM 880).

### 2.9 Western Blot Analysis

Rat colonic tissue (50 mg) or Caco‐2 cells (1 × 10^6^) were homogenized or lysed on ice for 30 min in radioimmunoprecipitation assay (RIPA) lysis buffer (Beyotime, Cat. No. P0013B) containing 1% PMSF and 1% phosphatase inhibitor cocktail. Total protein was obtained by centrifugation at 12,000 rpm for 15 min at 4°C. Cytoplasmic and nuclear protein fractions were extracted using a Nuclear and Cytoplasmic Protein Extraction Kit (Beyotime, Cat. No. P0027) according to the manufacturer's protocol. Protein concentrations were determined using a BCA assay kit (Thermo Scientific, Cat. No. 23225). Equal amounts of protein (30 μg) were resolved by SDS‐PAGE (sodium dodecyl sulfate‐polyacrylamide gel electrophoresis) and transferred onto PVDF (polyvinylidene fluoride) membranes (Millipore, Cat. No. IPVH00010). Membranes were blocked with 5% non‐fat milk for 1 h at room temperature and incubated overnight at 4°C with the following primary antibodies: ZO‐1 (Invitrogen, 40–2200, 1:1000), Occludin (Invitrogen, 71–1500, 1:1000), Claudin‐1 (Invitrogen, 51–9000, 1:1000), p‐BTK (Y223) (Cell Signaling Technology, 5082, 1:1000), p‐SYK (Cell Signaling Technology, 2710, 1:1000), p‐PLCγ2 (Cell Signaling Technology, 3874, 1:1000), p‐AKT (S473) (Cell Signaling Technology, 4060, 1:1000), IKK (Cell Signaling Technology, 2682, 1:1000), p‐IκBα (Cell Signaling Technology, 2859, 1:1000), cytoplasmic p65 (Cell Signaling Technology, 8242, 1:1000), nuclear p65 (Cell Signaling Technology, 6956, 1:1000), GAPDH (Cell Signaling Technology, 5174, 1:5000), and Histone H3 (Cell Signaling Technology, 4499, 1:1000). After washing, membranes were incubated with HRP‐conjugated goat anti‐rabbit secondary antibody (ZSGB‐BIO, Cat. No. ZB‐2301, 1:5000) for 1 h at room temperature. Protein bands were visualized using an ECL chemiluminescence kit (Millipore, Cat. No. WBKLS0500) and imaged with a ChemiDoc XRS+ system (Bio‐Rad). Band intensities were quantified using ImageJ software (v1.53t). Relative protein expression was normalized to GAPDH (total and cytoplasmic proteins) or Histone H3 (nuclear proteins).

### 2.10 Quantitative Real‐Time PCR (qPCR)

Total RNA was extracted from rat colonic tissue using TRIzol reagent (Invitrogen, Cat. No. 15596026) and quantified with a NanoDrop 2000 spectrophotometer. Reverse transcription was performed using the PrimeScript RT reagent kit (Takara, Cat. No. RR047A). qPCR was carried out on an ABI 7500 system with SYBR Premix Ex Taq II (Takara, Cat. No. RR820A) under the following cycling conditions: 95°C for 30 s, followed by 40 cycles of 95°C for 5 s and 60°C for 30 s. The following genes were analyzed: *BTK*, *SYK*, *PLCG2*, *PIK3CD*, *PIK3CA*, *AKT1*, and *NFKB1*. *GAPDH* (glyceraldehyde‐3‐phosphate dehydrogenase) served as the endogenous reference. Relative expression levels were calculated using the 2^(−ΔΔCt) method.

Primer sequences were as follows:

- *BTK*: forward 5′‐GTCAGAGACTCCAGCAAAGCTG‐3′, reverse 5′‐TACTGGCTCTGAGGTGTGGAAC‐3′;
- *SYK*: forward 5′‐CCTGCTGCTGATGATGGTGA‐3′, reverse 5′‐AGGTGGTCTCGATGGTGGTA‐3′;
- *PLCG2*: forward 5′‐CCGACTCTTACGCCATCA‐3′, reverse 5′‐GGGTAGCGAAGCCTCATC‐3′;
- *PIK3CD*: forward 5′‐AGCTGCTGCTGATGATGGTG‐3′, reverse 5′‐GGTGGTCTCGATGGTGGTAG‐3′;
- *PIK3CA*: forward 5′‐TGGCTATAAACGGGAACG‐3′, reverse 5′‐TGCCGAATTGCTAGGTAC‐3′;
- *AKT1*: forward 5′‐TACCTGAAGCTACTGGGCAAGGG‐3′, reverse 5′‐CGGTCGTGGGTCTGGAATGAG‐3′;
- *NFKB1*: forward 5′‐AGCCAGAGGTTATTCAGCCCT‐3′, reverse 5′‐GCAACTGGAAAGAGGCTGTC‐3′;
- *GAPDH*: forward 5′‐TGGTGCTGAGTATGTCGTGG‐3′, reverse 5′‐GGCGGAGATGATGACCCTTT‐3′.

### 2.11 Elisa

Colonic mucosal tissue (50 mg) was homogenized in ice‐cold PBS (pH 7.4) and centrifuged at 12,000 rpm for 10 min at 4°C to collect the supernatant. Rat serum samples and Caco‐2 cell culture supernatants were centrifuged at 3000 rpm for 10 min to remove cellular debris. Levels of rat TNF‐α (R&D Systems, Cat. No. RTA00), IL‐6 (R&D Systems, Cat. No. R6000B), IL‐1β (R&D Systems, Cat. No. RLB00), and MCP‐1 (R&D Systems, Cat. No. RMC00), as well as human TNF‐α (R&D Systems, Cat. No. DTA00C), IL‐6 (R&D Systems, Cat. No. D6050), IL‐1β (R&D Systems, Cat. No. DLB50), and MCP‐1 (R&D Systems, Cat. No. DCP00), were measured using commercially available ELISA kits strictly according to the manufacturer's protocols. Absorbance was measured at 450 nm using a multimode microplate reader (Tecan Infinite M200 Pro), and cytokine concentrations were calculated from standard curves.

### 2.12 Flow Cytometric Apoptosis Assay

Apoptosis of Caco‐2 cells was assessed using an Annexin V‐FITC/PI apoptosis detection kit (BD Biosciences, Cat. No. 556547). Following treatments, culture medium was discarded, and cells were washed twice with ice‐cold PBS. Cells were detached using 0.25% trypsin without EDTA (Gibco, Cat. No. 25200056) and adjusted to a density of 1 × 10^6^ cells/mL. Cells were incubated with 5 μL Annexin V‐FITC and 5 μL propidium iodide (PI) in the dark for 15 min. Samples were analyzed on a BD FACSCanto II flow cytometer, and apoptotic rates were quantified using FlowJo software.

### 2.13 Isolation of Colonic Mucosal Mononuclear Cells and Detection of CD19 ^+^ B Cells

Colonic mucosal tissue was minced and digested with collagenase type IV (Sigma, Cat. No. C5138) in the presence of DNase I (Thermo Scientific, Cat. No. EN0521). The resulting cell suspension was passed through a 70 μm cell strainer to obtain mononuclear cells. Cells were resuspended in flow cytometry staining buffer (BioLegend, Cat. No. 420201) and incubated with PE‐conjugated anti‐rat CD19 antibody (BioLegend, Cat. No. 202408, 1:200) for 30 min at 4°C in the dark. After filtration through flow cytometry tubes (BD, Cat. No. 352235), samples were acquired on a CytoFLEX flow cytometer (Beckman Coulter), and the proportion of CD19^+^ B cells was analyzed using CytExpert software.

### 2.14 Detection of CD63 ^+^ Mast Cells and CD69 ^+^ Lymphocytes

Supernatants and adherent cells from co‐culture systems were collected, mixed by gentle pipetting, and centrifuged. Cell pellets were incubated with PE‐conjugated anti‐human CD63 antibody (BioLegend, Cat. No. 353004, 1:200) and FITC‐conjugated anti‐human CD69 antibody (BioLegend, Cat. No. 310904, 1:200) for 30 min at 4°C in the dark. Residual erythrocytes were lysed using red blood cell lysis buffer (BD, Cat. No. 555899). Samples were analyzed by flow cytometry to quantify the proportions of CD63^+^ activated mast cells and CD69^+^ early activated lymphocytes.

### 2.15 Statistical Analysis

All experiments were independently repeated three times, and data are expressed as mean ± standard deviation (SD). Statistical analyses were performed using GraphPad Prism 9.0 software. Multi‐group comparisons were conducted by one‐way analysis of variance (ANOVA) followed by Tukey's post hoc test for pairwise comparisons. A *p* value < 0.05 was considered statistically significant.

## 3 Results

### 3.1 Polyphenol Composition and Systemic Bioavailability of TQDZD Constituents After Dietary Intake

Comprehensive qualitative analysis of Tiaoqi Daozhi Decoction (TQDZD) aqueous extract was performed using high‐resolution UPLC‐MS/MS in both positive and negative ion modes, yielding a total of 1962 non‐redundant compounds (Figure S1). Metabolite class composition revealed that the formula is predominantly constituted by phenolic acids, flavonoids, terpenoids, alkaloids, and amino acid derivatives, with phenolic acids and flavonoids representing the most abundant classes and thus forming the core chemical foundation of the formula (Figure 1A). These polyphenol‐rich profiles are characteristic of functional foods with gut‐protective potential. To delineate the nutritionally relevant material basis of TQDZD in vivo, we conducted a parallel comparative analysis of serum from three diet‐exposed rats, serum from three blank control rats, and the original decoction using the same UPLC‐MS/MS platform. By cross‐referencing the mass spectral features of the decoction with those of dosed and blank sera, a total of 257 systemically bioavailable components were identified, comprising 150 prototype constituents (58.4%) and 107 metabolites. The predominance of prototype constituents (> 50%) suggests that the functional effects of TQDZD are primarily mediated by the orally bioavailable parent compounds rather than by extensive biotransformation.

**FIGURE 1:**
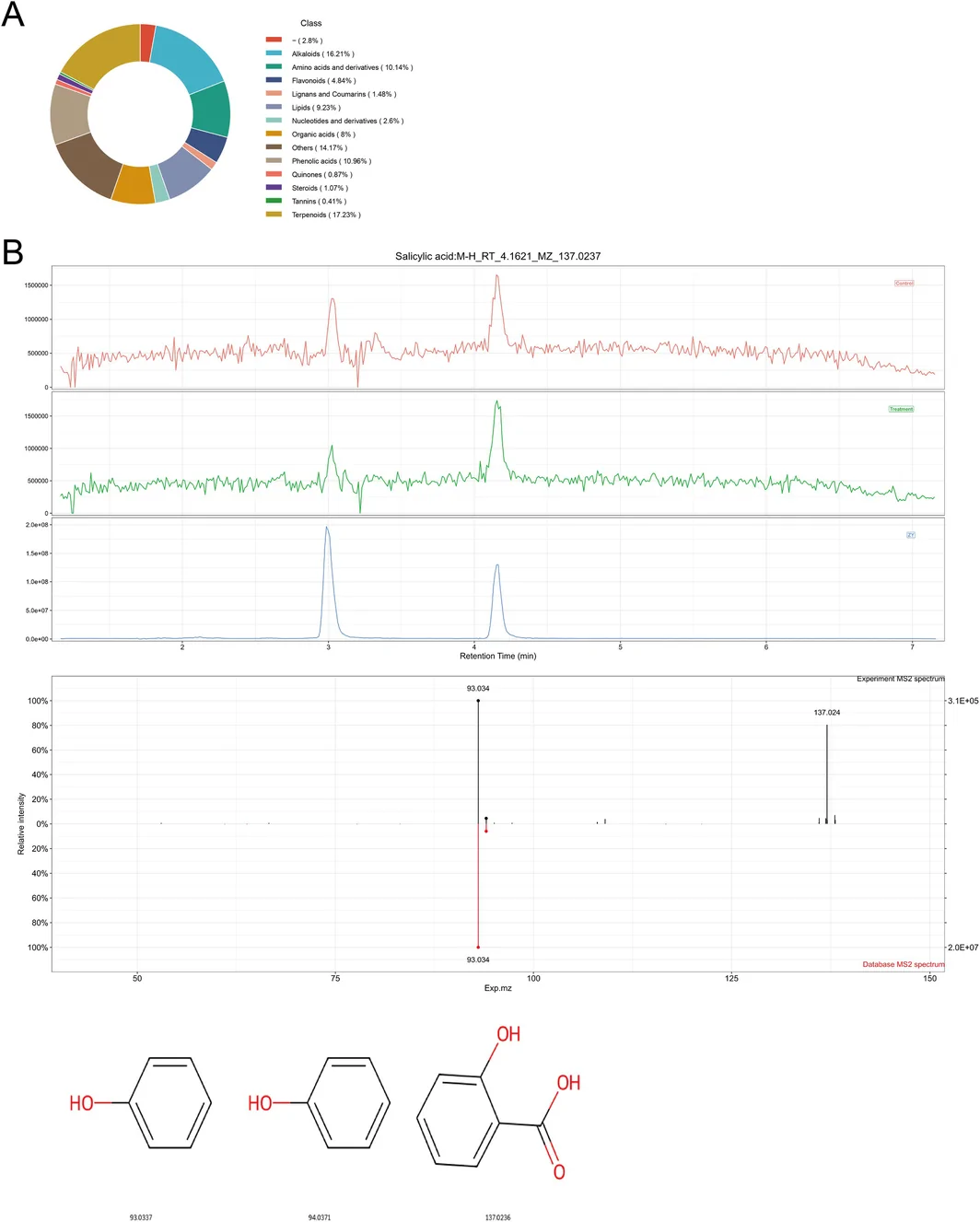
Chemical composition of Tiaoqi Daozhi Decoction and identification of the core systemically bioavailable prototype constituent. (A) Donut chart depicting the metabolite class composition of TQDZD aqueous extract. Phenolic acids and flavonoids constitute the predominant classes. (B) Extracted ion chromatogram (EIC) and MS/MS fragmentation spectrum of salicylic acid—the most abundant core systemically bioavailable constituent with high relevance to IBS‐C. Screening criteria: Treatment/Control peak area ratio ≥ 2; top‐20 abundance rank in the decoction; CV < 30% across three diet‐exposed serum replicates; Level 1a identification confidence (authentic standard match of retention time, precursor m/z, and MS/MS fragments). Level 1 (A) indicates confirmation via comparison with reference standards, i.e., retention time, primary molecular weight, and secondary fragment ions are fully matched with those of the reference standard. Level 1 (B) indicates confirmation without reference standards, where secondary fragment ions are fully matched with database entries. Both levels are based on high‐resolution mass spectrometry data and differ only in whether reference standards are used for verification.

To prioritize core systemically bioavailable constituents with potential nutritional bioactivity, we applied stringent multi‐dimensional screening criteria: (i) peak area ratio (Treatment vs. Control) ≥ 2; (ii) abundance ranking within the top 20 in the original decoction; (iii) coefficient of variation (CV) < 30% across three diet‐exposed serum replicates; and (iv) identification confidence Level 1a (authentic standard confirmed by matching retention time, precursor m/z, and MS/MS fragments) or Level 1b (no standard, but MS/MS fragments fully matched to spectral databases). Four core systemically bioavailable prototypes satisfied these criteria: salicylic acid, N‐acetyl‐L‐phenylalanine, L‐valyl‐L‐phenylalanine, and L‐phenylalanyl‐L‐hydroxyproline (Table 1). Among these, salicylic acid—a naturally occurring phenolic acid widely distributed in plant‐based foods—was prioritized for functional validation. All four compounds were markedly enriched in diet‐exposed serum relative to blank controls (ratio 2.05–10.36) and exhibited excellent reproducibility across the three parallel serum samples (CV < 30%).

**TABLE 1:** Identification of core blood‐absorbed prototype constituents from Tiaoqi Daozhi Decoction.

Compounds	Class I	Theoretical m/z	RT (min)	Adduct	Level	Score	Mean peak area (Serum)	Mean peak area (NC)	Ratio (Treatment/Control)
Salicylic acid	Phenolic acids	137.02439	4.1621	[M−H]−	1a	0.8358	17,972,737	8,763,957	2.0507559
N‐Acetyl‐L‐phenylalanine	Amino acids and derivatives	206.0822	3.7593	[M−H]−	1a	0.8682	16,293,415	7975901.3	2.0428305
L‐Valyl‐L‐phenylalanine	Amino acids and derivatives	264.14689	1.3841	[M+]+	1b	0.6781	11,696,481	3394436.9	3.4457795
Phe‐Hyp	Amino acids and derivatives	279.134	2.2332	[M+H]+	1a	0.9052	151437.64	14624.09	10.355355

To further ensure identification accuracy, extracted ion chromatograms (EIC) and MS/MS spectra of the four core constituents were obtained for triple confirmation based on retention time, accurate mass, and characteristic fragment ions (Figure 1B and Figure S2). The deviations between theoretical and measured m/z were consistently < 5 ppm, and MS/MS fragmentation patterns showed excellent concordance with authentic standards or spectral databases, validating the reliability of the identifications.

A total of 107 systemically bioavailable metabolites were identified, comprising primarily Phase I metabolites (oxidation, reduction, methylation) and Phase II conjugates (glucuronidation, sulfation). Notably, no corresponding metabolites were detected for any of the four core prototypes. This observation may reflect rapid metabolic clearance of these compounds, metabolite abundance below the analytical detection limit, or, more importantly, the possibility that these constituents circulate predominantly as intact parent molecules, thereby directly engaging their target organs to exert therapeutic effects.

### 3.2 Untargeted Serum Metabolomics Analysis

#### 3.2.1 Quality Control and Global Metabolic Profiling

To elucidate how systemically bioavailable bioactive constituents translate into systemic metabolic and physiological effects, we performed untargeted metabolomics analysis, focusing on the regulatory impact of TQDZD on the inflammation‐metabolism network associated with IBS‐C. Serum samples from normal control (NC), model (IBS‐C), and TQDZD‐treated groups were analyzed using high‐resolution UPLC‐MS/MS. A total of 2190 metabolites were detected, of which 1897 were confirmed by MS/MS (922 in positive ion mode, 975 in negative ion mode). Metabolite class composition analysis revealed that in negative ion mode, lipids and lipid‐like molecules were the most abundant class (37.78%), followed by organoheterocyclic compounds (16.77%) and organic acids and derivatives (15.27%); in positive ion mode, organoheterocyclic compounds (22.67%), lipids and lipid‐like molecules (20.26%), and organic acids and derivatives (19.29%) predominated (Figure 2A,B). The global metabolic profile comprehensively covered core metabolic categories intimately linked to intestinal function and inflammatory responses. Stringent quality assessment using quality control (QC) samples throughout the analytical run confirmed excellent reproducibility: total ion chromatogram (TIC) overlays of QC samples in both ionization modes demonstrated high concordance in retention time and peak intensity (Figure 2C,D); pairwise Pearson correlation coefficients among QC samples exceeded 0.92; and cumulative distribution analysis of coefficient of variation (CV) within biological replicates showed that > 75% of metabolites had CV < 0.3 and > 85% had CV < 0.5 (Figure 2E–H). These metrics fully satisfy international quality standards for untargeted metabolomics.

**FIGURE 2:**
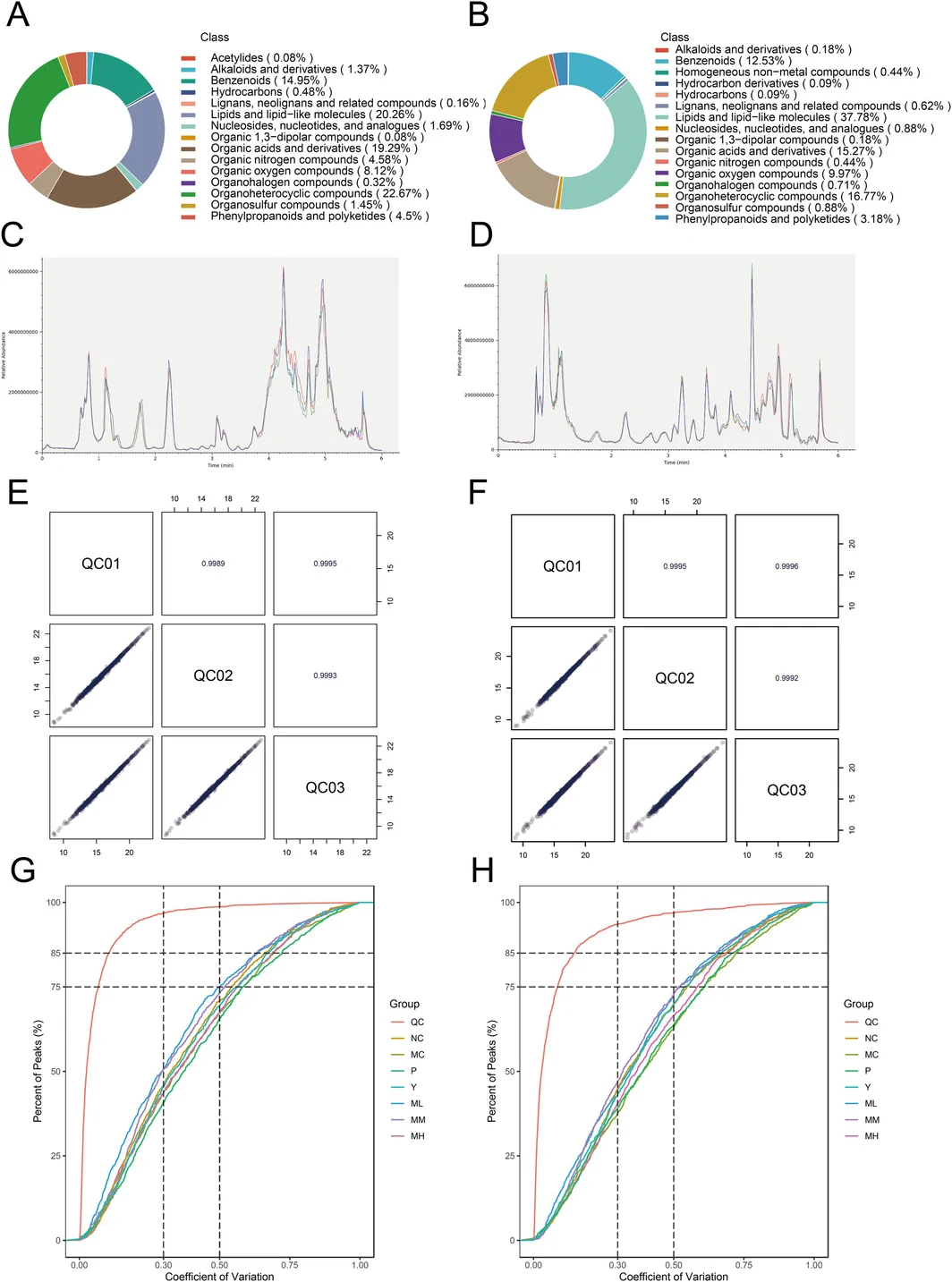
Quality assessment of untargeted metabolomics and metabolite class composition. (A) Donut chart showing serum metabolite class composition in positive ion mode. (B) Donut chart showing serum metabolite class composition in negative ion mode. (C) Overlaid total ion chromatograms (TIC) of quality control (QC) samples in positive ion mode, demonstrating excellent retention time and peak intensity reproducibility. (D) Overlaid TIC of QC samples in negative ion mode. (E) Correlation matrix of QC samples in positive ion mode. Diagonal tiles display QC sample identifiers; lower‐left tiles show scatter plots of log‐transformed metabolite intensities between corresponding QC pairs, with each point representing a metabolite; upper‐right tiles present Pearson correlation coefficients (all > 0.92). (F) Correlation matrix of QC samples in negative ion mode. (G) Cumulative distribution of coefficient of variation (CV) across experimental groups in positive ion mode. The x‐axis denotes CV value; the y‐axis denotes the proportion of metabolites with CV below the corresponding value. Different colors represent distinct groups. Vertical reference lines indicate CV = 0.3 and CV = 0.5; horizontal reference lines indicate the 75% and 85% metabolite proportion thresholds. (H) Cumulative CV distribution across groups in negative ion mode.

#### 3.2.2 Differential Metabolite Screening and OPLS‐DA Analysis

Orthogonal partial least‐squares discriminant analysis (OPLS‐DA) was applied to globally compare serum metabolic profiles across groups. Samples from the model group (MC) and normal control group (NC) separated completely in the OPLS‐DA score space, indicating pronounced alteration of the serum metabolome in IBS‐C rats (Figure 3A). The low‐ (ML), medium‐ (MM), and high‐dose (MH) TQDZD groups, as well as the reference compounds pinaverium bromide (P) and orelabrutinib (Y), all showed clear separation from the model group (Figure 3B–F). Inter‐dose comparisons revealed distinct metabolic divergence between the high‐dose group and the low‐/medium‐dose groups (Figure 3G–I), and multi‐group global comparisons further confirmed appreciable discrimination in metabolic features among groups (Figure 3J,K).

**FIGURE 3:**
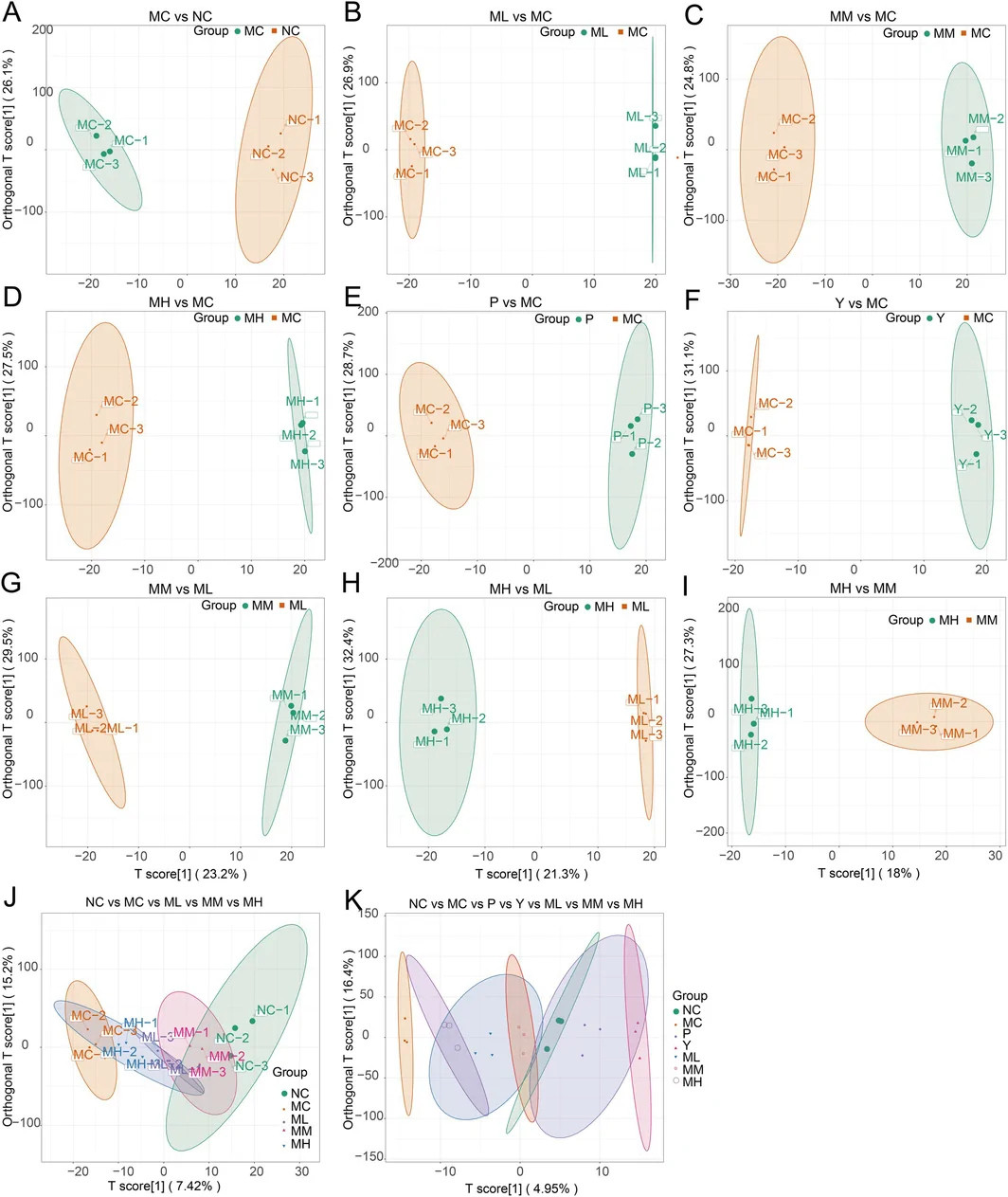
Orthogonal partial least‐squares discriminant analysis (OPLS‐DA) score plots. (A) Model (MC) vs. normal control (NC). (B) Low‐dose TQDZD (ML) vs. MC. (C) Medium‐dose TQDZD (MM) vs. MC. (D) High‐dose TQDZD (MH) vs. MC. (E) Pinaverium bromide (P) vs. MC. (F) Orelabrutinib (Y) vs. MC. (G) MM vs. ML. (H) MH vs. ML. (I) MH vs. MM. (J) Five‐group comparison: NC, MC, ML, MM, MH. (K) Seven‐group comparison: NC, MC, P, Y, ML, MM, MH. Each point represents an individual sample; samples from the same group are identically colored. The x‐axis (predictive component) captures inter‐group variation; the y‐axis (orthogonal component) captures intra‐group variation. Percentages in parentheses denote the proportion of variance explained by each component.

Differential metabolites were identified from OPLS‐DA models using thresholds of variable importance in projection (VIP) > 1 and adjusted *p* < 0.05 after Benjamini‐Hochberg (BH) correction. Induction of IBS‐C markedly altered the serum metabolic profile, yielding 49 differential metabolites (28 downregulated, 21 upregulated) relative to NC (Figure 4A). TQDZD intervention elicited dose‐dependent metabolomic shifts: compared with MC, 114, 126, and 101 differential metabolites were identified in the ML, MM, and MH groups, respectively (Figure 4B–D). Reference comparisons revealed 51 differential metabolites (30 down, 21 up) between the pinaverium bromide group (P) and MC, and 65 differential metabolites (46 down, 19 up) between the BTK modulator orelabrutinib group (Y) and MC (Figure 4E,F). Inter‐dose comparisons identified 78, 57, and 39 differential metabolites for MM vs. ML, MH vs. ML, and MH vs. MM, respectively (Figure 4G–I). Multi‐group global comparisons (NC vs. MC vs. ML vs. MM vs. MH; NC vs. MC vs. P vs. Y vs. ML vs. MM vs. MH) identified 115 and 105 differential metabolites, respectively, with no overt global trend in up‐ or downregulation.

**FIGURE 4:**
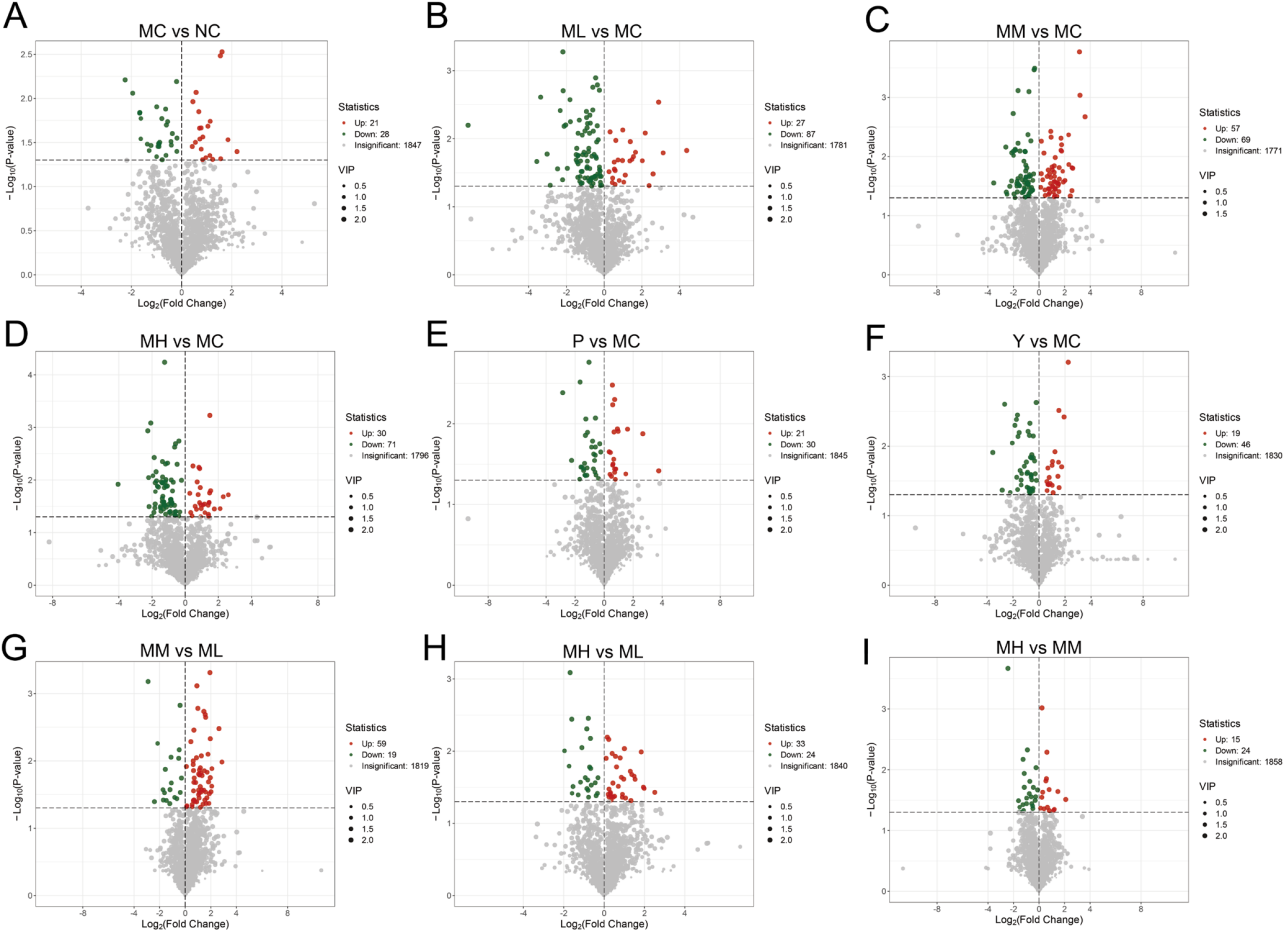
Volcano plots of differential metabolites. (A) MC vs. NC. (B) ML vs. MC. (C) MM vs. MC. (D) MH vs. MC. (E) P vs. MC. (F) Y vs. MC. (G) MM vs. ML. (H) MH vs. ML. (I) MH vs. MM. Each point represents a metabolite. Red, significantly upregulated; green, significantly downregulated; gray, detected but not statistically significant. Selection criteria: VIP > 1 from OPLS‐DA and *p* < 0.05 after Benjamini‐Hochberg (BH) correction. The x‐axis denotes log_2_ fold change (Log_2_FC); larger absolute values indicate greater inter‐group differences. The y‐axis denotes statistical significance (−Log_10_ *p*‐value). Dot size reflects VIP score.

#### 3.2.3 Hierarchical Clustering and Correlation Network Analysis of Differential Metabolites

Unsupervised two‐way hierarchical clustering was performed on differential metabolites across group comparisons, and heatmaps were generated (Figure 5A–I). All samples clustered clearly according to their experimental groups, with tight grouping of biological replicates and no apparent outliers, confirming the excellent reproducibility and reliability of the dataset. Based on HMDB secondary classification, lipids and lipid‐like molecules constituted the most abundant differential metabolite class across all pairwise comparisons, followed by organoheterocyclic compounds and organic acids and derivatives (Figure 5A–I). Coverage of metabolite classes varied among treatment groups: TQDZD medium‐ and high‐dose groups engaged additional classes including alkaloids and derivatives, organosulfur compounds, phenylpropanoids, and polyketides, whereas the pinaverium bromide group was restricted to only six fundamental classes. Heatmap expression patterns revealed clear discrimination between model and normal control metabolic profiles. TQDZD‐treated groups exhibited graded expression profiles in a dose‐dependent manner, with the high‐dose group displaying a pattern most closely resembling that of the BTK inhibitor orelabrutinib.

**FIGURE 5:**
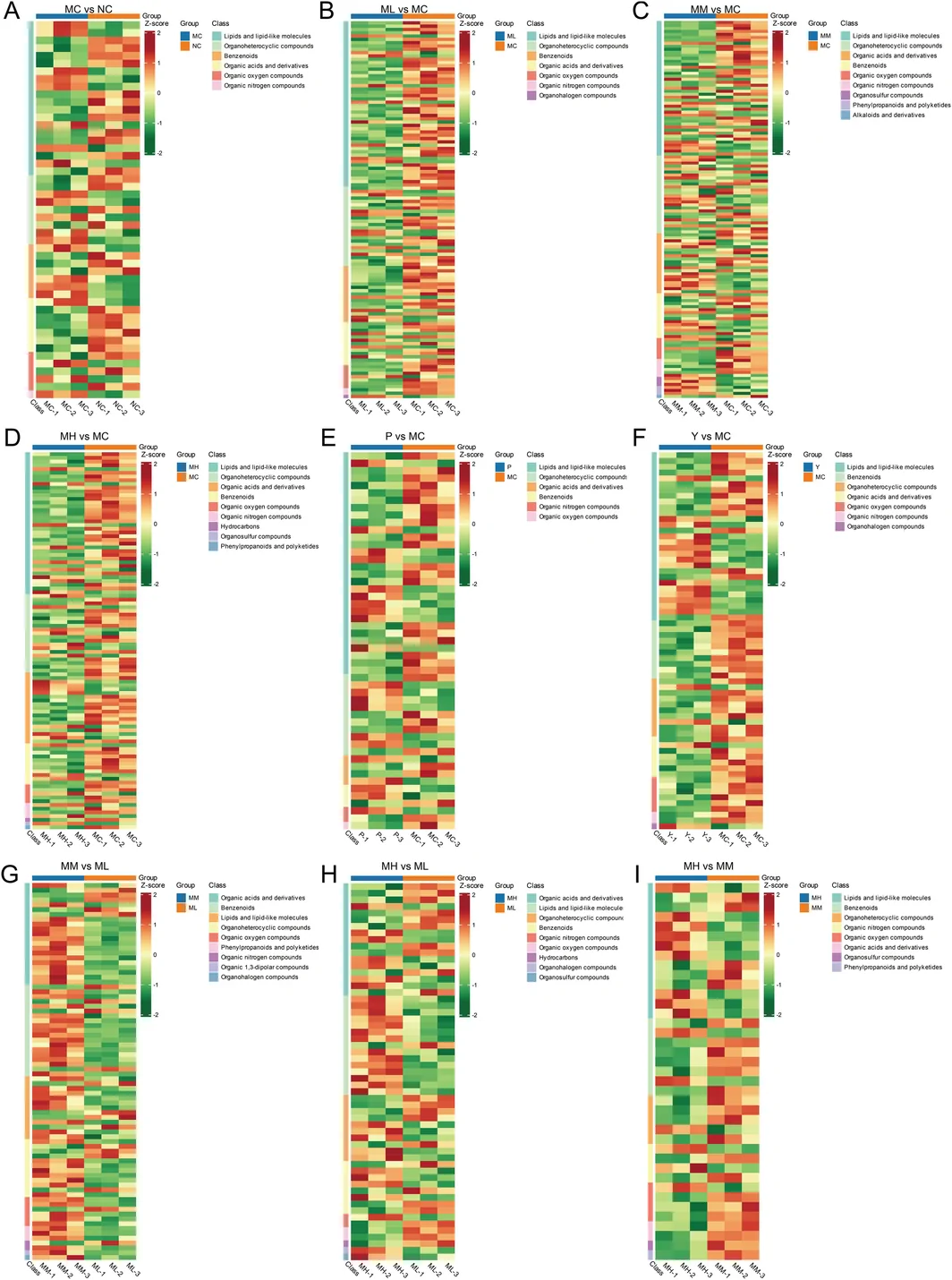
Heatmaps with hierarchical clustering of differential metabolites. (A) MC vs. NC. (B) ML vs. MC. (C) MM vs. MC. (D) MH vs. MC. (E) P vs. MC. (F) Y vs. MC. (G) MM vs. ML. (H) MH vs. ML. (I) MH vs. MM. Rows represent differential metabolites; columns represent individual samples grouped by condition. Colors indicate normalized metabolite abundance (red, high; green, low). Left dendrograms depict metabolite clustering; top dendrograms (where applicable) depict sample clustering. Metabolite class annotations follow HMDB secondary classification.

To probe coordinated regulatory relationships among differential metabolites, correlation networks were constructed for each comparison (Figure S3A–I). In the model group (MC vs. NC), a tightly interconnected module centered on glycerophospholipids, fatty acyls, and carboxylic acid derivatives emerged, with all intra‐module correlations being significantly positive (Figure S3A). TQDZD intervention remodeled this module in a dose‐dependent manner: in the low‐dose group (ML vs. MC), the core module composition was partially altered, with metabolites such as prenol lipids and indole derivatives joining the network (Figure S3B); in the medium‐dose group (MM vs. MC), the overall connectivity of the core module diminished further, and new metabolites including naphthalenes and heteroaromatic compounds became engaged (Figure S3C); in the high‐dose group (MH vs. MC), the original core module structure was substantially reorganized, with pyrrolidines and glycerolipids emerging as novel hub nodes (Figure S3D). Reference analyses revealed that the pinaverium bromide‐associated network (P vs. MC) was centered on tetrapyrrole and quinoline derivatives (Figure S3E), whereas the network structure of the orelabrutinib group (Y vs. MC) closely resembled that of high‐dose TQDZD, both featuring glycerophospholipids, prenol lipids, and carboxylic acid derivatives as principal connectivity nodes (Figure S3F). Inter‐dose comparisons showed that differentially correlated metabolites were primarily enriched in organic acids, phenylpropanoids, and lipid derivatives (Figure S3G–I). Collectively, these findings strongly implicate aberrant lipid metabolic networks in the pathogenesis of IBS‐C.

#### 3.2.4 KEGG Pathway Enrichment Analysis of Differential Metabolites

KEGG pathway enrichment analysis was performed for differential metabolites in each comparison, and the results are presented as bubble plots (Figure 6A–I). In IBS‐C model rats, differential serum metabolites were predominantly enriched in lipid metabolism and signal transduction pathways, including linoleic acid metabolism, sphingolipid metabolism, mTOR signaling, and primary bile acid biosynthesis (Figure 6A). TQDZD intervention dose‐dependently enriched inflammation‐associated lipid metabolic pathways: the low‐, medium‐, and high‐dose groups all exhibited significant enrichment of glycerophospholipid metabolism, arachidonic acid metabolism, linoleic acid metabolism, and C‐type lectin receptor signaling (Figure 6B–D). Notably, the high‐dose group additionally enriched innate immune‐related pathways such as necroptosis and Fcγ receptor‐mediated phagocytosis, and its enrichment profile closely mirrored that of the selective BTK modulator orelabrutinib (Figure 6F).

**FIGURE 6:**
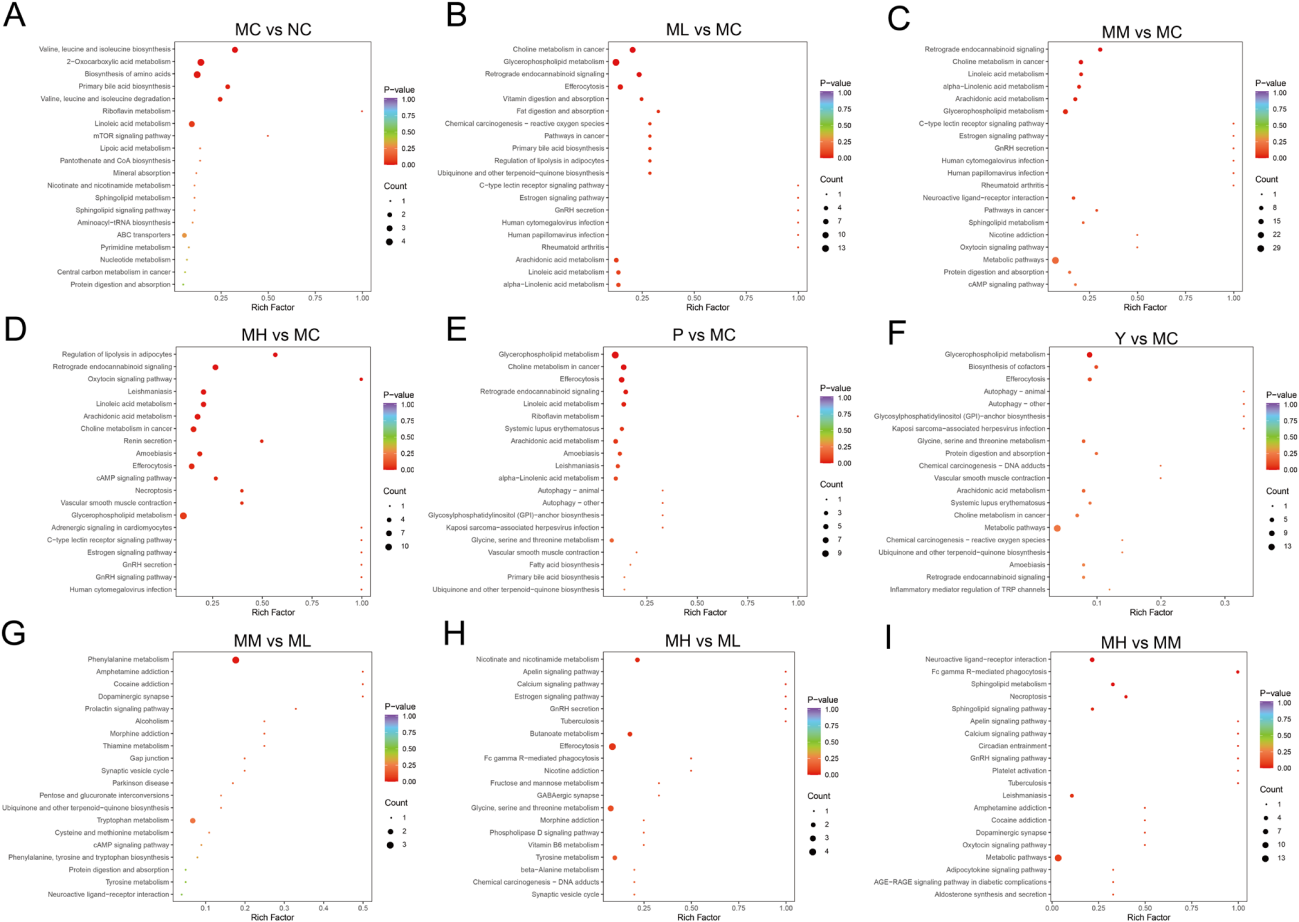
KEGG pathway enrichment bubble plots of differential metabolites. (A) MC vs. NC. (B) ML vs. MC. (C) MM vs. MC. (D) MH vs. MC. (E) P vs. MC. (F) Y vs. MC. (G) MM vs. ML. (H) MH vs. ML. (I) MH vs. MM. The x‐axis represents the Rich Factor for each pathway; the y‐axis lists pathway names ordered by *p*‐value. Dot color reflects *p*‐value magnitude (redder denotes greater enrichment significance). Dot size indicates the number of differential metabolites enriched in the pathway.

The pinaverium bromide group was primarily enriched in pathways related to primary bile acid biosynthesis, fatty acid biosynthesis, and vascular smooth muscle contraction, with markedly weaker enrichment of inflammation‐associated pathways compared with the high‐dose TQDZD and orelabrutinib groups (Figure 6E). Inter‐dose comparisons revealed that differential pathways among TQDZD dose groups were predominantly associated with neurotransmitter signaling and immunomodulation (Figure 6G–I). Collectively, untargeted metabolomics identified “lipid‐inflammatory pathway dysregulation” as a core metabolic signature of IBS‐C and demonstrated that TQDZD and the BTK modulator orelabrutinib elicit highly convergent metabolic remodeling. This finding strongly suggests that BTK‐linked inflammatory signaling may represent a key nutritional mechanism through which TQDZD ameliorates IBS‐C.

### 3.3 Tiaoqi Daozhi Decoction Improves Intestinal Epithelial Barrier Function and Suppresses Colonic Mucosal Inflammation in IBS‐C Rats

#### 3.3.1 TQDZD Restores Impaired Intestinal Epithelial Barrier Integrity

The metabolomics analyses described above indicated that aberrant lipid‐inflammatory pathways constitute a core metabolic feature of IBS‐C. We therefore examined the effects of TQDZD on intestinal barrier function and colonic mucosal inflammation in IBS‐C rats. Disruption of the intestinal epithelial barrier is a primary trigger of low‐grade mucosal inflammation in IBS‐C. Serum FITC‐dextran assays revealed that intestinal permeability was markedly elevated in the model group relative to normal controls (*p* < 0.001). TQDZD intervention reduced permeability in a dose‐dependent manner, with the high‐dose group achieving the most pronounced effect (*p* < 0.001), comparable to that of the BTK modulator reference orelabrutinib (Figure 7A). HE and PAS staining of colonic tissue confirmed that the model group exhibited epithelial desquamation, goblet cell depletion, and mild inflammatory infiltration. In contrast, the high‐dose TQDZD and orelabrutinib groups largely restored mucosal epithelial integrity and increased goblet cell numbers (Figure 7B). Both Western blot and immunofluorescence analyses consistently demonstrated that expression of the tight junction proteins ZO‐1, Occludin, and Claudin‐1 was significantly downregulated in the colonic mucosa of model rats (*p* < 0.001). TQDZD dose‐dependently upregulated the expression of these proteins (*p* < 0.05), with the high‐dose group outperforming the low‐ and medium‐dose groups (*p* < 0.05) (Figure 7C,D).

**FIGURE 7:**
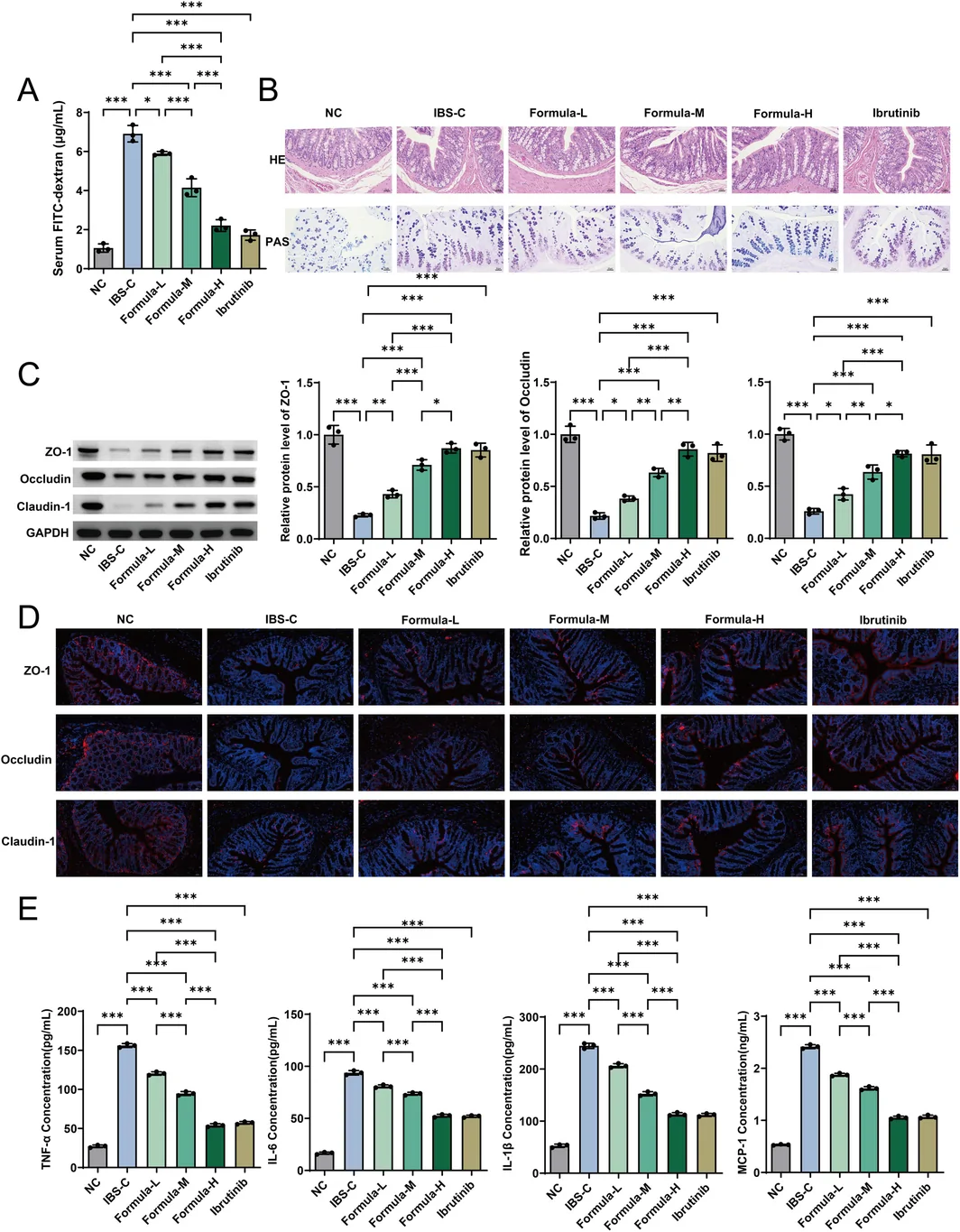
Tiaoqi Daozhi Decoction ameliorates intestinal epithelial barrier dysfunction and colonic mucosal inflammation in IBS‐C rats. (A) Serum FITC‐dextran permeability assay. (B) Representative HE and PAS staining of colonic tissue. HE staining reveals mucosal epithelial integrity and inflammatory infiltration; PAS staining highlights goblet cell abundance and mucus secretion. Scale bar = 50 μm. (C) Western blot analysis of tight junction protein (ZO‐1, Occludin, Claudin‐1) expression in colonic mucosa. (D) Immunofluorescence staining showing localization and expression of ZO‐1 (red), Occludin (red), and Claudin‐1 (red) in colonic tissue. Nuclei are counterstained with DAPI (blue). Scale bar = 50 μm. (E) ELISA quantification of pro‐inflammatory cytokines TNF‐α, IL‐6, IL‐1β, and chemokine MCP‐1 in colonic mucosal tissue. Data are presented as mean ± SEM. *p < 0.05, **p < 0.01, ***p < 0.001.

#### 3.3.2 TQDZD Suppresses Innate Immune Cell Activation and Pro‐Inflammatory Cytokine Release in the Colonic Mucosa

ELISA measurements revealed that levels of the pro‐inflammatory cytokines TNF‐α, IL‐6, and IL‐1β, as well as the chemokine MCP‐1, were markedly elevated in both colonic mucosa and serum of model rats relative to normal controls (*p* < 0.001). High‐dose TQDZD significantly reduced these inflammatory mediators (*p* < 0.001), achieving an inhibitory effect comparable to that of orelabrutinib (Figure 7E; Figure S4A).

Toluidine blue staining of mast cells, immunohistochemical analysis, and flow cytometric detection of CD19^+^ B cells demonstrated that the model group exhibited substantially increased infiltration of mast cells and B cells within the colonic mucosa, with the majority of mast cells displaying a degranulated phenotype. All TQDZD dose groups and the orelabrutinib group significantly attenuated the infiltration of these immune cells and suppressed mast cell degranulation (Figure S4B–D). To dissect the underlying nutritional mechanism, we examined key components of the NF‐κB signaling pathway. Western blot analysis revealed that phosphorylation levels of BTK, SYK, phospholipase Cγ2 (PLCγ2), protein kinase B (AKT), and downstream inhibitor of NF‐κB α (IκBα) were significantly elevated in the colonic mucosa of model rats, accompanied by decreased cytoplasmic p65 and pronounced p65 nuclear translocation (*p* < 0.001). TQDZD intervention dose‐dependently reversed the phosphorylation of these signaling molecules and attenuated p65 nuclear translocation, mirroring the regulatory pattern of orelabrutinib (Figure S4E). IκB kinase (IKK) expression remained unchanged across all groups. qPCR analysis further confirmed that mRNA expression of *BTK*, *SYK*, *PLCG2*, *PIK3CD*, *PIK3CA*, *AKT1*, and *NFKB1* was significantly upregulated in the colonic mucosa of model rats (*p* < 0.001), and these aberrant transcriptional changes were markedly reversed by high‐dose TQDZD and orelabrutinib (*p* < 0.001) (Figure S4F). Collectively, these in vivo findings demonstrate that TQDZD attenuates low‐grade colonic mucosal inflammation and restores the damaged intestinal epithelial barrier by modulating the BTK‐SYK‐NF‐κB signaling axis. However, whether TQDZD functionally engages BTK as a primary mechanistic node requires rigorous validation at the cellular level.

### 3.4 BTK Signaling Modulation Underlies the Anti‐Inflammatory and Barrier‐Protective Effects of TQDZD

To explore the mechanistic basis of the protective actions of TQDZD against IBS‐C, we employed siRNA‐mediated knockdown and plasmid‐mediated overexpression strategies as complementary pharmacological tools to assess the dependency of TQDZD functional activity on BTK in vitro. FITC‐dextran permeability assays demonstrated that inflammatory stimulation severely disrupted intestinal epithelial monolayer integrity, leading to markedly increased permeability (*p* < 0.001), whereas TQDZD treatment significantly reduced permeability (*p* < 0.001) (Figure 8A). Western blot analysis further confirmed that TQDZD substantially modulated the phosphorylation of BTK, SYK, PLCγ2, and AKT, blocked the downstream IκBα/p65 cascade, and suppressed p65 nuclear translocation (*p* < 0.01) (Figure 8B). Consequently, TQDZD upregulated the expression of tight junction proteins ZO‐1, Occludin, and Claudin‐1 (*p* < 0.001) (Figure 8C), reduced the release of IL‐1β, IL‐6, MCP‐1, and TNF‐α into the culture supernatant (*p* < 0.001) (Figure 8D), and attenuated inflammation‐induced apoptosis of intestinal epithelial cells (*p* < 0.001) (Figure 8E). Flow cytometric analysis of co‐culture systems revealed that LPS stimulation markedly increased the proportions of CD63^+^ activated mast cells and CD69^+^ early activated lymphocytes (*p* < 0.001), whereas TQDZD treatment significantly decreased the positivity rates of these immune cell activation markers (*p* < 0.001) (Figure 8F,G), indicating that the formula concurrently restrains excessive activation of both innate and adaptive immune compartments.

**FIGURE 8:**
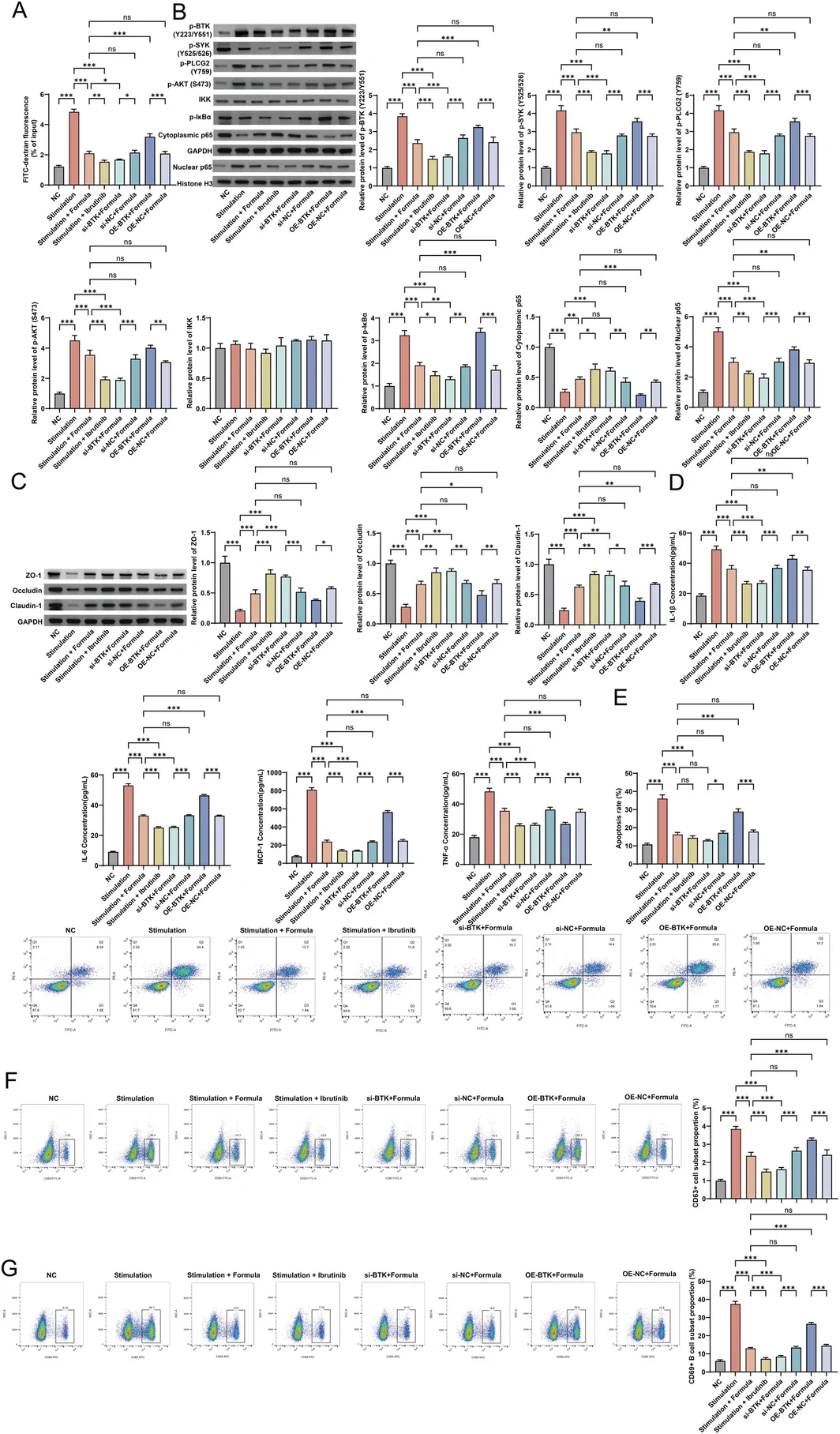
BTK is a critical target mediating the anti‐inflammatory and barrier‐protective effects of Tiaoqi Daozhi Decoction. (A) FITC‐dextran transepithelial permeability assay in Caco‐2 cell monolayers. (B) Western blot analysis of phosphorylation status of BTK‐SYK‐NF‐κB signaling components and p65 nuclear translocation. (C) Western blot analysis of tight junction protein (ZO‐1, Occludin, Claudin‐1) expression. (D) ELISA quantification of IL‐1β, IL‐6, MCP‐1, and TNF‐α in cell culture supernatants. (E) Flow cytometric assessment of apoptosis by Annexin V‐FITC/PI dual staining. Lower‐left quadrant (Annexin V^−^/PI^−^), viable cells; lower‐right quadrant (Annexin V^+^/PI^−^), early apoptotic cells; upper‐right quadrant (Annexin V^+^/PI^+^), late apoptotic/necrotic cells. Right panel shows quantification of total apoptotic rate (early + late). (F) Flow cytometric quantification of CD63^+^ activated mast cells in co‐culture systems. (G) Flow cytometric quantification of CD69^+^ early activated lymphocytes in co‐culture systems. *p < 0.05, **p < 0.01, ***p < 0.001.

BTK knockdown significantly potentiated the above protective actions of TQDZD. Compared with the si‐NC + TQDZD group, the si‐BTK + TQDZD group exhibited further reductions in intestinal epithelial permeability (*p* < 0.05), enhanced suppression of BTK, SYK, PLCγ2, and AKT phosphorylation, augmented blockade of the IκBα/p65 cascade and p65 nuclear translocation, lower pro‐inflammatory cytokine levels, and a further decline in apoptosis (*p* < 0.05) (Figure 8A–E). Moreover, the proportions of CD63^+^ mast cells and CD69^+^ lymphocytes were further decreased (*p* < 0.05) (Figure 8F,G). Conversely, BTK overexpression completely abrogated all protective effects of TQDZD, including barrier protection, inflammatory suppression, anti‐apoptotic activity, and inhibition of immune cell activation (Figure 8A–G). These consistent results indicate that BTK plays a critical role in the anti‐inflammatory, intestinal barrier‐protective, and anti‐apoptotic effects of TQDZD. The anti‐inflammatory effect of TQDZD is dependent on the BTK signaling pathway, which blocks downstream inflammatory signaling cascades and ultimately exerts a therapeutic effect on IBS‐C.

### 3.5 Salicylic Acid Is the Core Bioactive Constituent Responsible for the Therapeutic Efficacy of TQDZD

To identify the key active constituent within TQDZD that functionally engages BTK to elicit its protective effects, we performed systematic in vivo validation of salicylic acid, one of the core systemically bioavailable components identified in our earlier screening. Given that salicylic acid is a simple phenolic acid naturally present in a wide variety of edible plants, its identification as the principal bioactive component of TQDZD provides a mechanistic link between dietary phenolic acid intake and intestinal barrier protection.

Serum FITC‐dextran assays demonstrated that salicylic acid dose‐dependently reduced intestinal epithelial permeability in IBS‐C rats (*p* < 0.001) (Figure 9A). HE and PAS staining of colonic tissue confirmed that the high‐dose salicylic acid group substantially restored damaged mucosal epithelial integrity and increased goblet cell numbers (Figure 9B). Both Western blot and immunofluorescence analyses consistently showed that salicylic acid upregulated the expression of the tight junction proteins ZO‐1, Occludin, and Claudin‐1 in a dose‐dependent manner (Figure 9C,E). ELISA measurements further revealed that salicylic acid dose‐dependently decreased the levels of the pro‐inflammatory cytokines TNF‐α, IL‐6, and IL‐1β, as well as the chemokine MCP‐1, in both colonic mucosa and serum (*p* < 0.001) (Figure 9D; Figure S5A). Toluidine blue staining of mast cells, immunohistochemical analysis, and flow cytometric detection of CD19^+^ B cells demonstrated that salicylic acid significantly attenuated the infiltration of mast cells and B cells in the colonic mucosa and suppressed mast cell degranulation (Figure 9F; Figure S5B,C).

**FIGURE 9:**
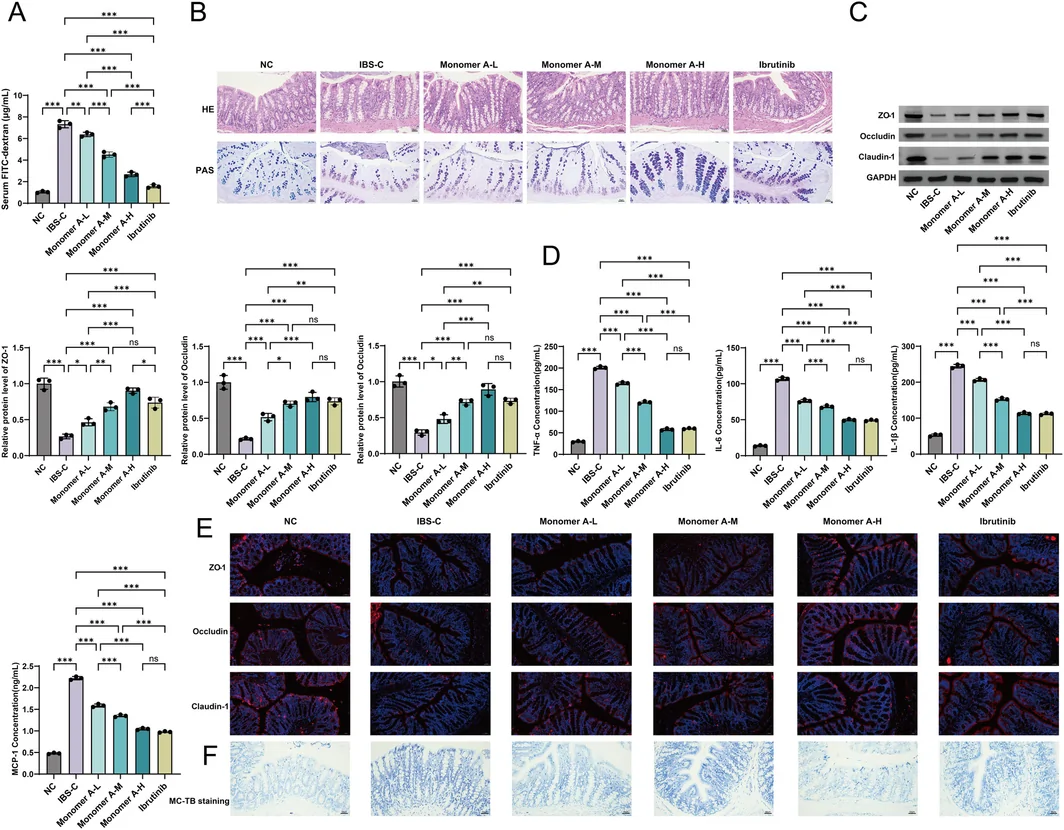
Salicylic acid ameliorates intestinal barrier dysfunction and colonic mucosal inflammation in IBS‐C rats. (A) Serum FITC‐dextran permeability assay. (B) Representative HE and PAS staining of colonic tissue, showing mucosal epithelial integrity, inflammatory infiltration, and goblet cell abundance. Scale bar = 50 μm. (C) Western blot analysis of tight junction protein (ZO‐1, Occludin, Claudin‐1) expression in colonic mucosa. (D) ELISA quantification of TNF‐α, IL‐6, IL‐1β, and MCP‐1 in colonic mucosal tissue. (E) Toluidine blue staining of mast cells in colonic mucosa, revealing infiltration and degranulation status. Scale bar = 50 μm. (F) Immunofluorescence staining showing localization and expression of ZO‐1 (red), Occludin (red), and Claudin‐1 (red) in colonic tissue. Nuclei are counterstained with DAPI (blue). Scale bar = 50 μm. Data are presented as mean ± SEM. *p < 0.05, **p < 0.01, ***p < 0.001.

Western blot and qPCR analyses further corroborated that salicylic acid dose‐dependently modulated the phosphorylation of BTK, SYK, PLCγ2, and AKT, blocked the downstream IκBα/p65 cascade and p65 nuclear translocation, and downregulated the mRNA expression of *BTK*, *SYK*, *PLCG2*, *PIK3CD*, *PIK3CA*, *AKT1*, and *NFKB1* (Figure S5D,E). These results demonstrate that salicylic acid is the core active constituent responsible for the anti‐inflammatory and intestinal barrier‐protective effects of TQDZD. Its anti‐inflammatory and barrier‐protective activities depend on the BTK signaling pathway, thereby achieving the therapeutic effect on IBS‐C.

### 3.6 BTK Signaling Is the Core Mechanistic Axis Mediating the Functional Actions of Salicylic Acid

To further determine whether the effects of salicylic acid are dependent on BTK, we employed an LPS‐induced intestinal epithelial cell inflammation model in conjunction with gene knockdown and overexpression approaches. FITC‐dextran permeability assays demonstrated that inflammatory stimulation severely compromised intestinal epithelial monolayer integrity, whereas salicylic acid treatment significantly reduced permeability (*p* < 0.001) (Figure 10A). Western blot analysis further confirmed that salicylic acid markedly modulated the phosphorylation of BTK, SYK, PLCγ2, and AKT, blocked the downstream IκBα/p65 cascade, and suppressed p65 nuclear translocation (*p* < 0.001) (Figure 10B). Consequently, salicylic acid upregulated the expression of the tight junction proteins ZO‐1, Occludin, and Claudin‐1 (*p* < 0.001) (Figure 10C), reduced the release of IL‐1β, IL‐6, MCP‐1, and TNF‐α into the culture supernatant (*p* < 0.001) (Figure 10D), and attenuated inflammation‐induced apoptosis of intestinal epithelial cells (*p* < 0.001) (Figure 10E).

**FIGURE 10:**
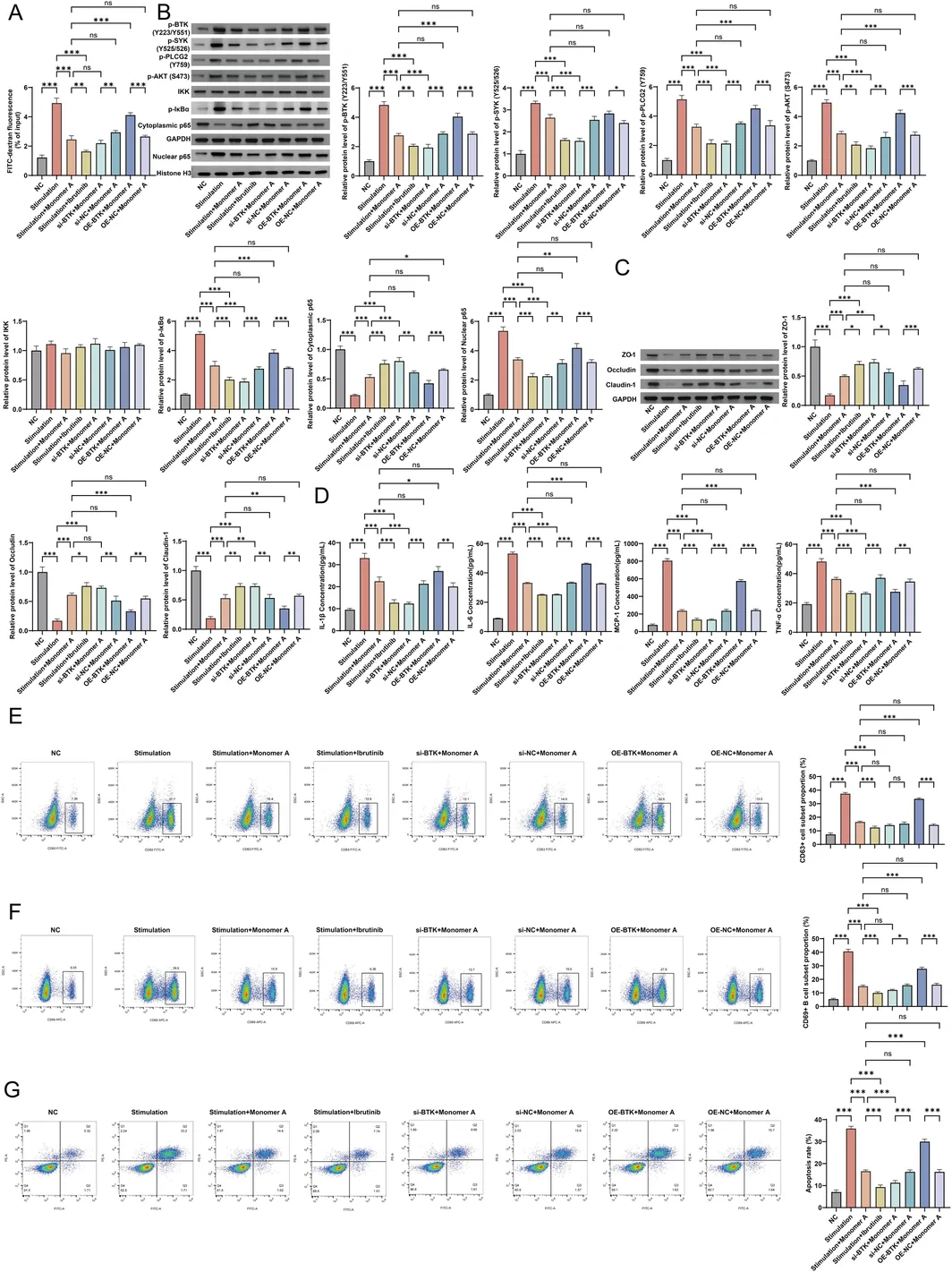
BTK is the core target mediating the anti‐inflammatory and barrier‐protective effects of salicylic acid (cellular validation). (A) FITC‐dextran transepithelial permeability assay in Caco‐2 monolayers. (B) Western blot analysis of phosphorylation status of BTK‐SYK‐NF‐κB signaling components and p65 nuclear translocation. (C) Western blot analysis of tight junction protein (ZO‐1, Occludin, Claudin‐1) expression. (D) ELISA quantification of IL‐1β, IL‐6, MCP‐1, and TNF‐α in cell culture supernatants. (E) Flow cytometric quantification of CD63^+^ activated mast cells in co‐culture systems. (F) Flow cytometric quantification of CD69^+^ activated lymphocytes in co‐culture systems. (G) Flow cytometric assessment of apoptosis. Data are presented as mean ± SEM. *p < 0.05, **p < 0.01, ***p < 0.001.

BTK knockdown significantly potentiated the above protective actions of salicylic acid. Compared with the si‐NC + salicylic acid group, the si‐BTK + salicylic acid group exhibited a further reduction in intestinal epithelial permeability (*p* < 0.01), enhanced suppression of the BTK‐SYK‐NF‐κB signaling axis, increased tight junction protein expression, lower pro‐inflammatory cytokine levels, and a further decline in apoptosis (*p* < 0.05) (Figure 10A–E). Conversely, BTK overexpression abrogated the protective actions of salicylic acid (Figure 10A–E). These consistent results confirm that the BTK signaling pathway plays a central role in mediating the anti‐inflammatory, intestinal barrier‐protective, and anti‐apoptotic effects of salicylic acid. The pharmacological action of salicylic acid relies on the BTK signaling pathway to block downstream inflammatory cascades, constituting the core pharmacodynamic material basis of TQDZD for IBS‐C treatment.

## 4 Discussion

From a functional food perspective, the present study addresses a critical gap in understanding how traditional medicine‐food homologous botanicals exert systemic metabolic effects through orally bioavailable polyphenols. While pharmacological studies often prioritize single‐target potency, dietary bioactive compounds such as phenolic acids are characterized by multi‐target, moderate‐affinity interactions that collectively remodel metabolic networks. The high proportion of prototype constituents detected in serum (> 58%) is particularly noteworthy from a nutritional standpoint, as it indicates favorable systemic exposure of intact bioactive molecules—a desirable property for functional food ingredients where consistent bioactivity across dietary intake is required.

The present study establishes a comprehensive research continuum encompassing global formula chemical profiling, systemically bioavailable bioactive constituent identification, serum metabolomic signature delineation, signaling pathway dissection, and key mechanistic node validation, thereby systematically elucidating both the nutritionally active material basis and the functional mechanism of Tiaoqi Daozhi Decoction in supporting intestinal homeostasis in constipation‐predominant irritable bowel syndrome. We identified salicylic acid as the core systemically bioavailable constituent of TQDZD. Its biological activity is dependent on the BTK signaling pathway, which blocks the downstream SYK‐NF‐κB inflammatory signaling cascade, thereby alleviating low‐grade colonic mucosal inflammation, repairing the damaged intestinal epithelial barrier, and ultimately exerting a therapeutic effect on IBS‐C. This discovery not only provides a robust scientific foundation for the traditional use of this medicine‐food homologous formula but also highlights BTK signaling as a nutritionally relevant molecular interface through which dietary bioactive compounds may exert their gut‐protective effects, offering a novel perspective for the development of functional food ingredients targeting intestinal health.

Metabolomic investigations have garnered considerable attention in the field of nutritional science, particularly for their capacity to reveal the systemic metabolic impact of dietary interventions (Kortesniemi et al. 2023; Wu et al. 2022). In the present study, untargeted serum metabolomics performed on normal control, model, and intervention groups yielded a total of 2190 detected metabolites, with lipids and lipid‐like molecules representing the most abundant class. OPLS‐DA analysis revealed complete separation between model and normal control samples, and the IBS‐C induced metabolic dysregulation was characterized by 49 differential metabolites. Hierarchical clustering and correlation network analysis uncovered a tightly interconnected module in the model group centered on glycerophospholipids, fatty acyls, and carboxylic acid derivatives, indicative of a highly coordinated lipid metabolic perturbation network in the IBS‐C state. KEGG enrichment analysis further demonstrated that differential metabolites were predominantly enriched in linoleic acid metabolism, sphingolipid metabolism, mTOR signaling, and primary bile acid biosynthesis. These findings align with prior reports documenting aberrant linoleic acid metabolism in IBS, and collectively delineate lipid‐inflammatory pathway dysregulation as a core metabolic signature of this condition (Hou et al. 2025; Stenlund et al. 2021). From a nutritional perspective, the observation that a botanical formula rich in phenolic acids and flavonoids can remodel this lipid‐centered metabolic network underscores the potential of food‐derived polyphenols to modulate systemic metabolic homeostasis, a property that holds considerable promise for dietary management of chronic gastrointestinal disorders.

Although the present study focused on host‐derived metabolic remodeling, the observed enrichment of glycerophospholipid and linoleic acid pathways is intimately linked to gut microbiota‐mediated polyphenol metabolism. Dietary phenolic acids are known to undergo extensive microbial biotransformation in the colon, producing metabolites with enhanced anti‐inflammatory activity (Russell et al. 2008; Kiriyama et al. 2024; Marhuenda‐Muñoz et al. 2019). The convergence of TQDZD‐induced metabolic trajectories with BTK‐linked lipid‐inflammatory networks suggests that host‐microbial co‐metabolism may amplify the functional effects of dietary salicylic acid. Future studies integrating metagenomic and metabolomic analyses will be essential to fully characterize the microbiota‐dependent bioactivation of TQDZD polyphenols.

The intervention with TQDZD remodeled the aforementioned lipid‐inflammatory network in a dose‐dependent manner, with the low, medium, and high dose groups all exhibiting significant enrichment of glycerophospholipid metabolism, arachidonic acid metabolism, linoleic acid metabolism, and C‐type lectin receptor signaling pathways. Critically, the metabolic regulatory pattern of the high dose TQDZD group closely resembled that of the selective BTK modulator orelabrutinib. Upon activation, BTK phosphorylates PLCγ2, which hydrolyzes phosphatidylinositol 4,5‐bisphosphate (PIP₂) to generate inositol 1,4,5‐trisphosphate (IP₃) and diacylglycerol (DAG), with DAG serving as a pivotal metabolic hub for both glycerophospholipid remodeling and arachidonic acid release. Thus, the functional convergence of TQDZD and orelabrutinib at the metabolomic level suggests that dietary phenolic acids and pharmacological BTK modulators may converge on common immunometabolic pathways, albeit through distinct modes of action—the former likely through allosteric or indirect metabolic modulation compatible with chronic dietary intake, the latter through direct enzymatic inhibition. This distinction is particularly relevant for functional food development: botanical mixtures with a high proportion of systemically absorbed prototype constituents may offer a more comprehensive and physiologically compatible metabolic regulatory profile compared to conventional pharmaceutical agents, which typically act through more restricted pathways. The high proportion of prototype constituents identified in TQDZD exceeding 50%, substantially higher than that reported for other herbal formulas, further supports the notion that this formulation exhibits favorable systemic exposure characteristics that enhance its predictability and controllability as a functional food candidate.

Notably, metabolomic analysis in this study revealed significant enrichment of lipid‐related pathways (glycerophospholipid metabolism and arachidonic acid metabolism), whereas BTK is primarily involved in immune regulation and intracellular signal transduction. The biological association between the two warrants further investigation. We propose the following hypothesis: lipid metabolic reprogramming can alter the cell membrane microenvironment, particularly the composition of membrane phospholipids and the level of phosphatidylinositol‐3,4,5‐trisphosphate (PIP3), thereby affecting the membrane localization, recruitment, and activation status of BTK (Shaw and Burke 2025; McAllister et al. 2025; Wang et al. 2019). As a key ligand for the pleckstrin homology (PH) domain of BTK, changes in the membrane abundance of PIP3 can directly regulate the membrane recruitment and kinase activation of BTK. Therefore, the remodeling of the lipid metabolic network by TQDZD may modulate the activation state of BTK by altering the membrane lipid microenvironment, thereby synergistically enhancing its inhibitory effect on the BTK signaling pathway. It should be emphasized that the mechanistic link described above is currently proposed only as a reasonable research hypothesis and remains to be validated by subsequent experiments, such as membrane lipidomics analysis and BTK membrane localization detection.

The pathogenesis of IBS involves multifactorial contributions, including impaired intestinal barrier function and immune cell activation (Kang et al. 2025; Cristina et al. 2012). Mild inflammatory responses are evident in the intestinal mucosa of IBS patients, with significant elevations in inflammatory mediators such as TNF‐α, IL‐6, and IL‐8, increased infiltration of immune cells including T cells, mast cells, and macrophages, and aberrant expression of tight junction proteins. Animal model studies have further demonstrated activation of the Toll‐like receptor 4 (TLR4)‐NF‐κB pathway in the colons of IBS‐D rats, leading to upregulation of IL‐8 and TNF‐α, whereas inhibition of this pathway reduces these inflammatory cytokines and improves mucosal architecture. These observations suggest that, although IBS is classified as a functional bowel disorder, persistent low‐grade inflammation and barrier disruption within the intestinal microenvironment may amplify symptomatology through inflammatory signaling cascades. The focal signaling axis identified in this study is the BTK‐SYK‐PLCγ2‐AKT‐NF‐κB cascade. In immune signaling, non‐receptor tyrosine kinases such as BTK and SYK play pivotal roles across diverse immune cell types. BTK participates not only in B cell receptor signaling but also in Toll‐like receptor and Fc receptor pathways, thereby modulating cytokine production. SYK is likewise expressed in various intestinal cell types, including intestinal epithelial cells, macrophages, dendritic cells, and mast cells, and its activation promotes the generation and secretion of pro‐inflammatory cytokines. Consequently, upstream activation of the BTK‐SYK axis invariably triggers downstream NF‐κB signaling cascades, further amplifying inflammatory responses. In colonic inflammatory disorders such as inflammatory bowel disease (IBD) and IBS, NF‐κB functions as a master transcription factor whose activation induces the expression of a plethora of pro‐inflammatory molecules and chemokines, exacerbating intestinal barrier damage and immune activation. The present study likewise observed marked upregulation of NF‐κB downstream effectors, including TNF‐α, IL‐6, IL‐1β, and MCP‐1, in the colons of IBS‐C model rats, indicative of NF‐κB pathway activation. It should be noted that the regulatory relationship between AKT and IκBα phosphorylation in the signaling pathway proposed in this study requires further clarification. It is known that IκBα phosphorylation is primarily mediated by the IKK complex, and AKT may indirectly promote IκBα phosphorylation by activating IKKα/β (Ozes et al. 1999; Sizemore et al. 2002; Dan et al. 2008). Since this study only detected the total protein expression level of IKK but not its phosphorylation status, the specific molecular mechanism from AKT to IκBα remains to be verified by further studies. Therefore, this signaling pathway should not be regarded as a fully confirmed cascade.

The results of this study demonstrate that TQDZD intervention dose‐dependently modulates the phosphorylation of BTK, SYK, PLCγ2, and AKT, blocks the downstream IκBα‐p65 cascade, and ultimately suppresses NF‐κB nuclear translocation, thereby markedly reducing the expression of pro‐inflammatory cytokines and restoring intestinal epithelial barrier function. This mechanism of action closely mirrors that of BTK modulators, yet with the distinct advantage that TQDZD is derived from medicine‐food homologous botanicals with a long history of safe consumption. Previous reports have shown that the BTK modulator orelabrutinib alleviates colonic inflammation in part through suppression of the phosphoinositide 3‐kinase (PI3K)‐Akt and NF‐κB signaling pathways. In the present study, we observed that TQDZD exhibits a metabolic profile highly similar to that of orelabrutinib, with both enriching pathways related to innate immunity‐associated lipid metabolism and Fcγ receptor signaling (Yu et al. 2025). Such pronounced convergence in metabolic regulatory patterns further substantiates the hypothesis that BTK signaling serves as a key nutritional target for TQDZD. At the molecular level, BTK knockdown significantly potentiated the anti‐inflammatory, barrier‐protective, and anti‐apoptotic effects of TQDZD, whereas BTK overexpression abrogated the beneficial actions of the formula, confirming the essential role of BTK signaling in mediating its functional activity. Although gene knockdown and overexpression experiments strongly support the critical role of BTK in the pharmacological effects of TQDZD, this study has not yet fully established a causal relationship through target‐specific inhibitor rescue assays. Current evidence supports a close correlation between the BTK signaling pathway and the therapeutic efficacy of TQDZD, and its full validation as a mechanistic causal relationship requires further investigation.

Through screening of systemically bioavailable components, we identified salicylic acid as one of the core bioactive molecules. Salicylic acid, which constitutes the core scaffold of aspirin and 5‐aminosalicylic acid, possesses well‐documented anti‐inflammatory properties. Studies have demonstrated that 5‐aminosalicylic acid can directly inhibit IKKα kinase activity, thereby blocking IκBα phosphorylation and suppressing NF‐κB activation (Yan and Polk 1999), and similarly, aspirin and sodium salicylate can inhibit IKKβ to impede NF‐κB activation (Mao et al. 2025). We observed that salicylic acid monotherapy also dose‐dependently reduced intestinal permeability, decreased pro‐inflammatory cytokines, and upregulated the expression of tight junction proteins (ZO‐1, Occludin, Claudin‐1) in IBS‐C rats, with a mechanism consistent with that of TQDZD. This further confirms that the anti‐inflammatory effect of salicylic acid is dependent on the BTK signaling pathway.

Importantly, salicylic acid is not unique to TQDZD but is ubiquitous in the human diet, occurring in fruits, vegetables, herbs, and spices at varying concentrations (Malakar et al. 2017). The doses used in the present study fall within the range achievable through polyphenol‐rich dietary patterns or traditional botanical beverage consumption. This positions TQDZD not as a source of novel pharmaceutical salicylates, but as a concentrated, standardized delivery system for naturally occurring dietary phenolic acids with established safety profiles and centuries of consumption history.

From a translational perspective, the identification of salicylic acid as a principal active component of TQDZD is particularly significant for functional food development. Phenolic acid‐rich botanical extracts are increasingly incorporated into functional beverages, dietary supplements, and fortified foods aimed at gut health (Wu et al. 2023; Kumar et al. 2025). The present findings provide a mechanistic rationale for such applications: by modulating BTK‐linked inflammatory metabolism, salicylic acid‐rich formulations may offer a dietary strategy for managing barrier‐centric gastrointestinal disorders without the systemic immunosuppression associated with pharmaceutical BTK inhibitors. Future food engineering approaches should focus on optimizing the bioaccessibility, sensory properties, and formulation stability of phenolic‐acid‐rich botanical extracts to enhance their practical applicability as functional food ingredients.

Despite the important advances made in this study, several limitations should be acknowledged. First, the functional effects of TQDZD and salicylic acid were characterized in a rat model of IBS‐C. While this provides proof‐of‐concept for the functional food potential, human dietary intervention trials are required to establish efficacy within the context of realistic dietary intake patterns and to determine optimal delivery formats (e.g., brewed decoctions, encapsulated extracts, or fortified foods). Second, the precise molecular interaction mode between salicylic acid and BTK remains to be fully characterized. However, for dietary bioactive compounds, functional efficacy does not necessarily require direct high‐affinity enzymatic inhibition; allosteric modulation, metabolite‐mediated indirect effects, or gut microbiota‐dependent bioactivation are equally plausible mechanisms that align with the multi‐target nature of food‐derived polyphenols. Third, only the function of salicylic acid was validated among the four core constituents; the roles of the remaining three core components and their potential synergistic interactions with salicylic acid remain to be elucidated. Exploring these synergistic interactions is particularly relevant for functional food science, as botanical formulations typically exert superior effects compared to isolated single compounds due to their multi‐component, multi‐target regulatory nature. Fourth, while the present study focused on host epithelial barrier and inflammatory signaling, the gut microbiota represents a critical yet unexplored dimension of TQDZD's functional activity. Dietary polyphenols are well‐established prebiotic‐like modulators that selectively enrich beneficial taxa and enhance short‐chain fatty acid production. Beyond traditional delivery formats, integrating TQDZD with emerging smart biomaterials and biosensing technologies offers new translational opportunities: stimuli‐responsive carriers could enable targeted delivery of salicylic acid in response to intestinal inflammatory cues, while flexible pH microsensors may facilitate real‐time monitoring of local microenvironmental changes during dietary intervention (Lan et al. 2026; Pan et al. 2026). Fifth, this study did not detect the phosphorylation levels of IKKα/β; therefore, the specific regulatory mechanism between AKT and IκBα phosphorylation (whether mediated by IKK) has not been fully elucidated. Integrating metagenomic, metabolomic, and gut‐brain axis assessments in future studies will provide a more comprehensive understanding of how this medicine‐food homologous formula exerts its systemic benefits through host‐microbial co‐metabolism. Nevertheless, the present work establishes a robust scientific foundation for the development of TQDZD as a functional food product targeting intestinal health. By elucidating the mechanism of action of its core constituent salicylic acid through BTK‐linked inflammatory signaling, this study bridges the gap between traditional dietary wisdom and modern nutritional science, offering both mechanistic insight and practical guidance for the rational design of food‐based interventions for chronic gastrointestinal disorders.

## 5 Conclusions

In summary, this study systematically elucidates the systemically bioactive constituents and nutritional mechanisms of Tiaoqi Daozhi Decoction in supporting intestinal homeostasis in constipation‐predominant irritable bowel syndrome, identifying salicylic acid as the core dietary bioactive component and confirming the critical role of the BTK signaling pathway in its pharmacological effects. These findings establish a robust scientific foundation for the traditional use of this medicine‐food homologous botanical formula and demonstrate that a phenolic acid naturally present in edible plants can modulate intestinal inflammatory signaling through a defined pathway, thereby providing mechanistic support for developing food‐based interventions that leverage the medicine‐food homology paradigm to bridge traditional dietary wisdom with modern nutritional science. Future investigations will encompass human dietary intervention studies to evaluate the efficacy of TQDZD and salicylic acid as part of a polyphenol‐rich dietary pattern in IBS‐C populations; food engineering approaches to optimize the bioaccessibility, sensory properties, and formulation stability of phenolic‐acid‐rich botanical extracts; systematic exploration of the remaining core constituents and their synergistic interactions to fully characterize the multi‐component regulatory network inherent to botanical functional foods; and integration of gut microbiota and gut‐brain axis dimensions through multi‐omics approaches to further refine the mechanistic framework underlying the host‐microbial‐metabolic interplay. Collectively, this work establishes a scientific paradigm for translating traditional botanical mixtures into evidence‐based functional foods, offering both mechanistic insight and practical guidance for harnessing edible plants in the dietary management of chronic gastrointestinal disorders.

## Author Contributions

**Min Duan:** conceptualization, methodology, investigation, data curation, formal analysis, visualization, writing – original draft. **Lixia Huang:** investigation, data curation, methodology, validation. **Yingyue Xu:** conceptualization, supervision, funding acquisition, project administration, writing – review and editing. **Ya Huang:** methodology, resources, supervision, project administration. **Xiaojun Yang:** conceptualization, supervision, funding acquisition, project administration, writing – review and editing.

## Funding

This work was supported by the Chongqing Medical Leading Talent Project (Grant No. YXLJ202409), the Chongqing Jiangbei District Science and Health Joint Medical Research Project (Grant No. 2025JBKWLH046), and the Organized Research Plan of Chongqing Traditional Chinese Medicine Hospital (Grant No. 2024YZZKY003).

## Ethics Statement

The study was conducted in accordance with the Declaration of Helsinki and approved by the Institutional Animal Care and Use Committee (IACUC) of Southwest University (approval number: IACUC‐20260312‐13).

## Conflicts of Interest

The authors declare no conflicts of interest.

## Supporting information


**Figure S1:** Representative total ion chromatograms (TIC) of Tiaoqi Daozhi Decoction extract and rat serum in positive and negative ion modes. (A) TIC of normal control (NC) serum in positive ion mode. (B) TIC of NC serum in negative ion mode. (C) TIC of Tiaoqi Daozhi Decoction aqueous extract (Drug) in positive ion mode. (D) TIC of Drug in negative ion mode. (E) TIC of dosed rat serum (Serum) in positive ion mode. (F) TIC of Serum in negative ion mode. All samples were analyzed using a high‐resolution UPLC‐MS/MS platform.
**Figure S2:** Extracted ion chromatograms (EIC) and MS/MS fragmentation spectra of the three additional core blood‐absorbed prototype constituents. (A) EIC and MS/MS spectrum of *N*‐acetyl‐L‐phenylalanine. (B) EIC and MS/MS spectrum of L‐valyl‐L‐phenylalanine. (C) EIC and MS/MS spectrum of L‐phenylalanyl‐L‐hydroxyproline. All constituents satisfy the same stringent multi‐dimensional screening criteria as described in Figure 1B, with MS/MS fragments fully matched to spectral databases (Level 1b).
**Figure S3:** Correlation network analysis of differential metabolites. Networks are shown for pairwise comparisons: (A) MC vs. NC. (B) ML vs. MC. (C) MM vs. MC. (D) MH vs. MC. (E) P vs. MC. (F) Y vs. MC. (G) MM vs. ML. (H) MH vs. ML. (I) MH vs. MM. Nodes represent individual differential metabolites; edges represent significant correlations.
**Figure S4:** Tiaoqi Daozhi Decoction suppresses innate immune cell activation and BTK‐SYK‐NF‐κB signaling in the colonic mucosa of IBS‐C rats. (A) ELISA quantification of TNF‐α, IL‐6, IL‐1β, and MCP‐1 in serum. (B) Toluidine blue staining of colonic mucosa showing mast cell infiltration and degranulation. Scale bar = 50 μm. (C) Flow cytometric quantification of CD19^+^ B cells among mononuclear cells isolated from rat colonic mucosa. (D) Immunohistochemical staining for the mast cell marker TPSAB1 and the B cell marker CD19 in colonic tissue. Brown staining indicates positive signal. Scale bar = 50 μm. (E) Western blot analysis of phosphorylation status of key BTK‐SYK‐NF‐κB signaling components and p65 nuclear translocation. Analytes: p‐BTK (Y223), p‐SYK, p‐PLCγ2, p‐AKT (S473), IKK, p‐IκBα, cytoplasmic p65, and nuclear p65. Histone H3 served as nuclear loading control; GAPDH served as cytoplasmic loading control. (F) qPCR analysis of relative mRNA expression of *BTK*, *SYK*, *PLCG2*, *PIK3CD*, *PIK3CA*, *AKT1*, and *NFKB1* in colonic mucosa. Data are presented as mean ± SEM. *p < 0.05, **p < 0.01, ***p < 0.001.
**Figure S5:** Salicylic acid regulates serum inflammatory cytokines, immune cell infiltration, and BTK signaling in IBS‐C rats. (A) ELISA quantification of TNF‐α, IL‐6, IL‐1β, and MCP‐1 in serum. (B) Immunohistochemical staining for TPSAB1 (mast cell marker) and CD19 (B cell marker) in colonic tissue. Brown staining indicates positive signal. Scale bar = 50 μm. (C) Flow cytometric quantification of CD19^+^ B cells among mononuclear cells isolated from rat colonic mucosa. (D) Western blot analysis of phosphorylation status of BTK‐SYK‐NF‐κB signaling proteins and p65 nuclear translocation. (E) qPCR analysis of relative mRNA expression of *BTK*, *SYK*, *PLCG2*, *PIK3CD*, *PIK3CA*, *AKT1*, and *NFKB1* in colonic mucosa. Data are presented as mean ± SEM. *p < 0.05, **p < 0.01, ***p < 0.001.

## Data Availability

The data that support the findings of this study are available from the corresponding author upon reasonable request.
