# Supplementary material for: Salicylic Acid as a Food‐Derived Bioactive Constituent of Tiaoqi Daozhi Decoction: A Potential Functional Food Ingredient to Restoring Gut Barrier Function in Constipation‐Predominant IBS
Source: Food Sci Nutr. 2026 Oct 26;14(11):e72483. doi: 10.1002/fsn3.72483 (PMC13710674; doi:10.1002/fsn3.72483)
Supplement: Supplementary file 1 [file FSN3-14-e72483-s001.docx]

**Supplementary Figure**

**Figure S1. Representative total ion chromatograms (TIC) of Tiaoqi Daozhi Decoction extract and rat serum in positive and negative ion modes.** (A) TIC of normal control (NC) serum in positive ion mode. (B) TIC of NC serum in negative ion mode. (C) TIC of Tiaoqi Daozhi Decoction aqueous extract (Drug) in positive ion mode. (D) TIC of Drug in negative ion mode. (E) TIC of dosed rat serum (Serum) in positive ion mode. (F) TIC of Serum in negative ion mode. All samples were analyzed using a high‑resolution UPLC‑MS/MS platform.

**Figure S2. Extracted ion chromatograms (EIC) and MS/MS fragmentation spectra of the three additional core blood‑absorbed prototype constituents.** (A) EIC and MS/MS spectrum of *N*‑acetyl‑L‑phenylalanine. (B) EIC and MS/MS spectrum of L‑valyl‑L‑phenylalanine. (C) EIC and MS/MS spectrum of L‑phenylalanyl‑L‑hydroxyproline. All constituents satisfy the same stringent multi‑dimensional screening criteria as described in Figure 1B, with MS/MS fragments fully matched to spectral databases (Level 1b).

**Figure S3. Correlation network analysis of differential metabolites.** Networks are shown for pairwise comparisons: (A) MC *vs.* NC. (B) ML *vs.* MC. (C) MM *vs.* MC. (D) MH *vs.* MC. (E) P *vs.* MC. (F) Y *vs.* MC. (G) MM *vs.* ML. (H) MH *vs.* ML. (I) MH *vs.* MM. Nodes represent individual differential metabolites; edges represent significant correlations.

**Figure S4. Tiaoqi Daozhi Decoction suppresses innate immune cell activation and BTK‑SYK‑NF‑κB signaling in the colonic mucosa of IBS‑C rats.** (A) ELISA quantification of TNF‑α, IL‑6, IL‑1β, and MCP‑1 in serum. (B) Toluidine blue staining of colonic mucosa showing mast cell infiltration and degranulation. Scale bar = 50 μm. (C) Flow cytometric quantification of CD19⁺ B cells among mononuclear cells isolated from rat colonic mucosa. (D) Immunohistochemical staining for the mast cell marker TPSAB1 and the B cell marker CD19 in colonic tissue. Brown staining indicates positive signal. Scale bar = 50 μm. (E) Western blot analysis of phosphorylation status of key BTK‑SYK‑NF‑κB signaling components and p65 nuclear translocation. Analytes: p‑BTK (Y223), p‑SYK, p‑PLCγ2, p‑AKT (S473), IKK, p‑IκBα, cytoplasmic p65, and nuclear p65. Histone H3 served as nuclear loading control; GAPDH served as cytoplasmic loading control. (F) qPCR analysis of relative mRNA expression of *BTK*, *SYK*, *PLCG2*, *PIK3CD*, *PIK3CA*, *AKT1*, and *NFKB1* in colonic mucosa. Data are presented as mean ± SEM. **P < 0.05, **P < 0.01, ***P < 0.001.*

**Figure S5. Salicylic acid regulates serum inflammatory cytokines, immune cell infiltration, and BTK signaling in IBS‑C rats.** (A) ELISA quantification of TNF‑α, IL‑6, IL‑1β, and MCP‑1 in serum. (B) Immunohistochemical staining for TPSAB1 (mast cell marker) and CD19 (B cell marker) in colonic tissue. Brown staining indicates positive signal. Scale bar = 50 μm. (C) Flow cytometric quantification of CD19⁺ B cells among mononuclear cells isolated from rat colonic mucosa. (D) Western blot analysis of phosphorylation status of BTK‑SYK‑NF‑κB signaling proteins and p65 nuclear translocation. (E) qPCR analysis of relative mRNA expression of *BTK*, *SYK*, *PLCG2*, *PIK3CD*, *PIK3CA*, *AKT1*, and *NFKB1* in colonic mucosa. Data are presented as mean ± SEM. **P < 0.05, **P < 0.01, ***P < 0.001.*
